# Adaptation of the CUGH global health competency framework in the Chinese context: a mixed-methods study

**DOI:** 10.1186/s41256-023-00327-w

**Published:** 2023-11-02

**Authors:** Wei Ding, Yayi Guan, Bernadette Peterhans, Axel Hoffmann, Xiao-Nong Zhou

**Affiliations:** 1 National Institute of Parasitic Diseases, Chinese Center for Disease Control and Prevention (Chinese Center for Tropical Diseases Research), NHC Key Laboratory of Parasite and Vector Biology, WHO Collaborating Centre for Tropical Diseases, National Center for International Research On Tropical Diseases, 207 Rui Jin Er Road, Shanghai, 200025 China; 2 Swiss Tropical and Public Health Institute, Basel, Switzerland; 3 University of Basel, Basel, Switzerland

**Keywords:** CUGH, Global health, Competency framework, China, Localization, Delphi

## Abstract

**Background:**

In 2014, the Consortium of Universities for Global Health (CUGH) developed a global health competency framework and called for its validation. Given China's increasing engagement in global health over the past decade, there is a need for a tailored competency framework to enhance the capacity of its workforce. This study aimed to localize the CUGH global health framework within the Chinese context, offering guidance to public health professionals in China to bolster their capabilities for international endeavors.

**Methods:**

Employing a modified Delphi consultation approach, this study adapted the CUGH global health competency framework through three consultation rounds and a panel discussion. A questionnaire employing a five-point Likert scale was developed to gather opinions from 37 experts on the significance and feasibility of each competency within the Chinese setting. Profiling information, judgment criteria, and familiarity with each competency were collected to assess experts' authority levels. Furthermore, a priority survey was administered to 51 experts to identify key competencies and provide recommendations for bolstering the capabilities of China's public health professionals. Data analysis was performed using Microsoft Excel.

**Results:**

The adapted framework comprises 10 domains and 37 competencies including: 1. Global Burden of Disease; 2. Social-economic, Environmental and Behavioral Determinants of Health; 3. The Impact of Globalization on Population Health, Health Systems, and Healthcare; 4. Major Global health initiatives and efforts; 5. Ethics, Health Equity and Social Justice; 6. Sociocultural, Political Awareness and Policy Promotion; 7. Personal Competencies and Professional Practice; 8. Capacity strengthening; 9. Collaboration, Partnering and Communication; 10. Programme Management. The priority survey underscored Domain 9, 10, and 4 as the foremost concern for Chinese public health professionals, urging active learning, critical thinking, open communication, experiential learning, and case-based studies. Institutions were advised to enhance their capacity, foster partnerships, and discern China's distinct role in the global health arena.

**Conclusions:**

This study adapted the CUGH framework within the Chinese context, evaluating the significance and feasibility of each competency. The adapted framework can serve as a tool for developing global health curricula and delineating roles for Chinese public health professionals. To ensure contextual compatibility, testing of the framework with diverse public health professionals is recommended, enabling precise refinement of competencies based on empirical results.

**Supplementary Information:**

The online version contains supplementary material available at 10.1186/s41256-023-00327-w.

## Background

The Consortium of Universities for Global Health (CUGH), a US based university network, developed a global health competency framework in 2014 through a multi-phased consultation process that engaged a diverse panel of experts with interdisciplinary backgrounds [1, 2]. CUGH's framework serves as a comprehensive guide to global health competencies, offering a general overview for "global citizens" pursuing various fields related to global health and a more specialized "program-oriented operational level" framework for individuals planning to dedicate a portion of their careers to global health endeavors. For global citizens, the framework identifies 13 competencies distributed across 8 domains, encompassing key areas such as the global burden of disease, the globalization of health and healthcare, social and environmental determinants of health, collaboration, partnering, and communication, ethics, professional practice, health equity and social justice, as well as sociocultural and political awareness. In addition to these 8 domains, the "program-oriented operational level" includes an extra 3 domains, totaling 11 domains in this category. These additional domains focus on capacity strengthening, program management, and strategic analysis [2].

Over the past decade, China has significantly intensified its involvement in global health initiatives [3–5]. At the national level, China has committed itself to advancing the United Nations' 2030 Sustainable Development Goals through collaborative efforts with other developing nations. It has established its International Development Cooperation Agency (CIDCA), initiated its first multilateral fund, the South-South Cooperation Assistance Fund (SSCAF), and engaged in multilateral dialogues with African and Southeast Asian countries through the Forums on China-Africa Cooperation (FOCAC) and the Lancang-Mekong Cooperation (LMC). Notably, in the Beijing Declaration of the Ministerial Forum of China-Africa Health Development in 2013, China underscored the importance of cooperation to support African countries' healthcare priorities. At the institutional level, public health organizations such as the Chinese Center for Disease Control and Prevention (China CDC) have actively participated in international public health emergencies, providing support, for instance, in building laboratory capacity during the Ebola outbreak in Sierra Leone. China has also undertaken overseas public health projects since 2014, including initiatives like the China-Zanzibar Cooperation Project for Schistosomiasis Control, maternal health projects in Ethiopia and Myanmar, the China-UK-Tanzania Pilot Project on Malaria Control, and the Australia-China-Papua New Guinea Pilot Cooperation on Malaria Control Project [6–9]. Public health professionals from China's disease control system form a significant part of the workforce involved in these global health projects. However, concerns have arisen regarding the readiness of these professionals for global health missions, as many lack prior international experience [10]. To address this issue, institutions have implemented on-the-job training programs to equip them with the necessary knowledge and skills for overseas work. Nevertheless, there remains a critical gap in the absence of a comprehensive global health competency framework that can guide curriculum development, continuous professional development assessments, and job function definitions for Chinese public health professionals. This gap may lead to inconsistencies between training programs and the desired competencies for the global health workforce [11].

The authors of the CUGH global health competency framework have emphasized the need for validating these competencies and conducting further research to determine the most effective strategies for incorporating them into educational programs. This study aims to localize the CUGH framework within the Chinese context by evaluating the significance and feasibility of each competency. Additionally, it seeks to offer recommendations for individuals and institutions in China to enhance their capacity in the field of global health.

## Methods

### Study design

**Fig. 1:**
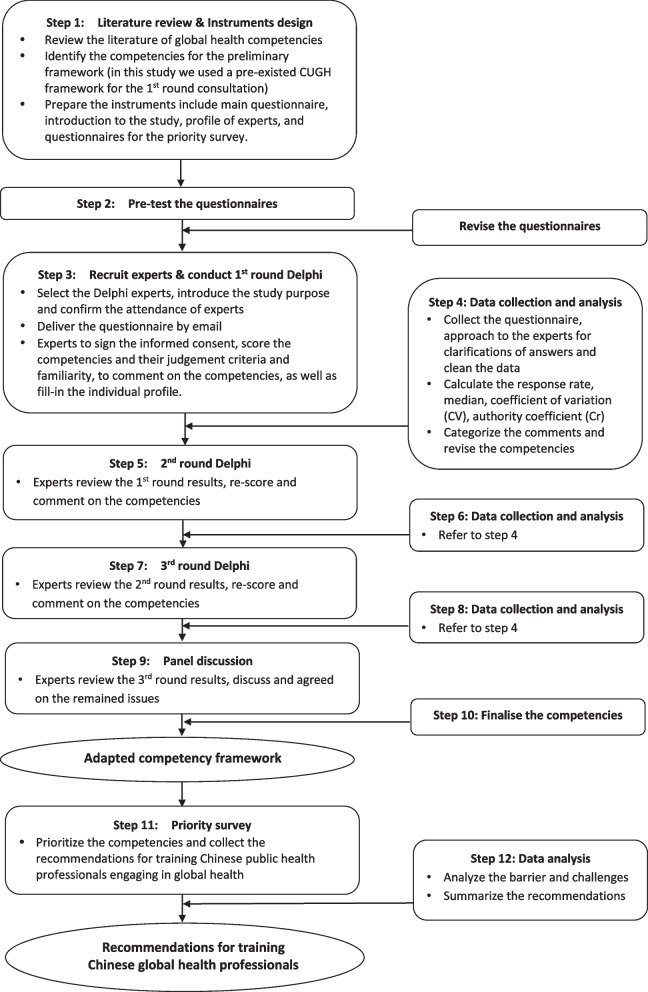
Flowchart of the study design

Figure 1 illustrates the two stages of the study. The first stage (step 1–10), initiated in August 2018, aimed to adapt the CUGH global health competency framework to the Chinese context using a modified Delphi consultation approach involving a panel of global health experts in China. The Delphi technique, originally developed by the Rand Corporation in the 1950s, is a widely employed method that employs structured questionnaires to facilitate group consensus among experts [12, 13]. The results of each questionnaire inform the design of the subsequent one, gradually leading to a higher level of consensus until a final agreement is achieved [14, 15]. In the concluding phase of this modified Delphi consultation, a panel discussion was convened with a smaller group of Delphi experts to address any remaining comments from the third round of consultation.

The second stage (step 11–12), which commenced in July 2019, focused on prioritizing the adapted competencies and gathering insights for the enhancement of educational programs catering to Chinese public health professionals. This stage involved a priority survey administered within an expanded pool of experts.

The Delphi panel comprised 53 individuals, with 30 experts selected from the pool of 69 members associated with the Chinese Society of Global Health and the Chinese Preventive Medicine Association (www.csgh.org.cn). An additional 23 experts were chosen through snowball or purposive sampling methods. To qualify as an expert, individuals were required to meet at least one of the following criteria: having a minimum of 5 years of work experience in global health or possessing a minimum of 5 years of work experience in public health with active engagement in global health activities within the past 2 years. The selection process also prioritized diversity in professional backgrounds to ensure the interdisciplinary nature of the Delphi panel. For the panel discussion aimed at finalizing the framework, a smaller group of 6 experts was chosen based on the following criteria: senior experts with more than 20 years of experience in either public or global health; active participation in all Delphi rounds; willingness to address any remaining comments; and representation from a variety of institutions and sectors.

The priority survey involved 37 experts who had completed the Delphi consultation, supplemented by an additional group of 24 experts. This additional group comprised 9 Chinese experts and 15 international experts, with the aim of obtaining a diverse range of perspectives. The selection of the 24 additional experts was carried out through purposive or snowball sampling methods. For Chinese experts, eligibility criteria followed those of the Delphi panel. In contrast, international experts were required to meet specific criteria: they must have a minimum of 5 years of work experience in global health and have collaborated with Chinese global health professionals for a minimum of 1 year. This criterion ensured that international experts possessed an understanding of the unique characteristics of Chinese global health professionals.

### Instrument development

The study employed two main instruments:

1. The Delphi Questionnaire (Additional file 1): This instrument included the study introduction, informed consent form, instructions for completing the questionnaire, the main questionnaire containing information for evaluating experts' authority, and experts' profiles.

The main questionnaire for the Delphi consultation was constructed based on the “program-oriented operational level” competencies outlined in the CUGH framework. This alignment was chosen to cater to the training needs of Chinese public health professionals who aim to spend a moderate amount of time working in the field of global health. Each competency was assessed in terms of two key dimensions:

- Significance, which gauged the importance of the competency within the Chinese context, and
- Feasibility, which determined the extent to which the competency could be objectively evaluated.

To measure these aspects, a five-point Likert scale was employed, ranging from 0 to 5 (0 = No significance or feasibility, 1 = Very low significance or feasibility, 2 = Low significance or feasibility, 3 = Moderate significance or feasibility, 4 = High significance or feasibility, 5 = Very high significance or feasibility). The questionnaire was prepared in Microsoft Word, with each competency translated into Chinese. The original CUGH framework was included for reference by the experts.

2. Questionnaire for the Priority Survey (Additional files 2 and 3): This questionnaire was developed using the internet-based survey tool WJX (www.wjx.cn) and offered in two language versions (Chinese and English). Following the completion of the Delphi consultation, it comprised three sections: (1) Identification of Priority Competency Domains and Rationale for Selection; (2) Advice for Advancing Global Health Education; and (3) Profiles of the Experts.

Prior to implementation, all instruments underwent pre-testing and revision processes to ensure their effectiveness and clarity.

### Study procedure

In the 1st round of consultation, we established contact with the selected 53 experts through email or instant messaging platforms, providing them with a comprehensive overview of the study's objectives and procedures. Those who expressed willingness to participate were requested to complete a consent form, as well as the questionnaire and an individual profile. Experts were encouraged to offer insights by adding, revising, removing, or commenting on the competencies outlined. Upon receiving the completed questionnaires, we incorporated the experts' feedback to refine the competencies. Competencies meeting the inclusion criteria progressed to the subsequent round of consultation. During the 2nd round consultation, the experts were asked to review, re-score and comment on the updated competencies. Corresponding revisions were made and prompted the 3rd round consultation. Following the third round, we organized a panel discussion, engaging a smaller group of experts, to reach a final consensus on the competencies. The outcomes of this panel discussion were then communicated to all participating experts.

For the priority survey, we distributed the questionnaire to the Delphi experts via email or instant messaging, depending on their preferred mode of communication, and accompanied it with a brief introduction to the survey's purpose. Simultaneously, we reached out to the 9 additional Chinese experts and 15 international experts via email, introducing the study and seeking their participation. Those who consented to take part received a second email containing the link to access the survey on the WJX platform. The survey responses were automatically collected through the WJX system.

### Data process and analysis

In line with other Delphi studies [16], the inclusion criteria of each competency was were defined as follows: a competency was considered included if it received a score of ≥ 3 from over 70% of the participants. Median was calculated to describe the central tendency of experts’ responses [17]. Coefficient of variation (CV) was used to describe the dispersals of experts’ responses. Authority coefficient (C_r_) was used to assess the degree of each expert’s authority in relation to their judgment criterion and familiarity when evaluating the indicators [18]. C_r_ is defined as: C_r_ = (C_a_ + C_s_)/2 (C_a_ refers to the experts’ judgment criterion, and C_s_ denotes the experts’ familiarity to each indicator, values of C_a_ and C_s_ are listed in Additional file 4). Results of each round Delphi consultation were double-entered and analyzed using Microsoft Excel 2010. Prior to making revisions, the experts' comments were thoroughly reviewed and scrutinized.

Regarding the priority survey, quantitative data were directly analyzed using the WJX platform. Qualitative data were systematically categorized, coded, and summarized within Microsoft Excel 2010. Recommendations were subsequently derived from the survey results.

## Results

### Basic information of the Delphi experts

The characteristics of the participating experts and their response rates are presented in Additional file 5. Out of the 53 experts contacted, a commendable 48 responded to the consultation, with 37 experts completing all three rounds of consultation. These experts possessed diverse professional backgrounds, with the majority in public health (73%), followed by global health (42%), health management or health policy (15%), clinical medicine (8%), development studies (4%), and diplomacy or international politics (4%). Their affiliations spanned various sectors, including universities (44%), disease control departments (27%), government and its affiliated institutions (15%), enterprises (6%), international organizations (4%), and non-governmental organizations (4%). Among them, 58.33% and 22.92% held senior and sub-senior professional titles, while 18.75% held intermediate titles. 75% of the experts boasted over 5 years of experience in global health, with 43.75% having accumulated 10 or more years in the field.

The average authoritative coefficients (C_r_) across the three rounds of consultation stood at 0.82, signifying a moderate to high degree of expertise among the experts (Table 1).

**Table 1:** Levels of authority of the Delphi experts

Round	Judgment criterion (C_a_)	Familiarity (C_s_)	Authority (C_r_)*
1st round	0.89	0.69	0.79
2nd round	0.90	0.73	0.85
3rd round	0.89	0.77	0.83
Average	0.89	0.73	0.82

*C_r_ = (C_a_ + C_s_)/2

### Delphi consultation round 1–3

The 1st round Delphi questionnaire encompassed 11 competency domains and 39 secondary competencies, with detailed results and revisions provided in Additional file 6. The experts' scores indicated a strong central tendency concerning the significance and feasibility of these competencies, demonstrating low dispersion. Median scores for competency significance and feasibility ranged from 4 to 5 and from 3 to 5, respectively. Mean scores ranged from 4.02 to 4.87 for significance and from 3.31 to 4.50 for feasibility. CVs for competency significance and feasibility ranged from 0.07 to 0.23 and from 0.15 to 0.32, respectively, indicating low dispersion levels. No competency met the criteria for removal. Experts' feedback underscored the need for clearer definitions of Chinese public health professionals, the avoidance of overlaps among secondary competencies, and the enhancement of coherence among domains.

The 2nd round Delphi questionnaire incorporated 13 competency domains and 52 secondary competencies, with detailed results and revisions provided in Additional file 7. Scores in this round demonstrated a slightly stronger central tendency for the competencies. Median scores for competency significance and feasibility ranged from 4 to 5 and from 3.5 to 5, respectively. Mean scores ranged from 4.03 to 4.88 for significance and from 3.58 to 4.75 for feasibility. CVs for competency significance and feasibility ranged from 0.07 to 0.20 and from 0.10 to 0.27, respectively, indicating lower dispersion levels than the 1st round. No competency met the criteria for removal. Experts reached a consensus to further modify domain structures by minimizing overlaps, reordering competencies to enhance coherence, and providing clarifications for competencies requiring further definition.

The 3rd round Delphi questionnaire included 11 competency domains and 42 secondary competencies, with detailed results available in Additional file 8. Scores in this round revealed a relatively strong central tendency for competency importance and feasibility. Median scores for significance and feasibility ranged from 4 to 5 and from 3.5 to 5, respectively. Mean scores ranged from 3.80 to 4.89 for significance and from 3.54 to 4.55 for feasibility. CVs for competency significance and feasibility ranged from 0.07 to 0.21 and from 0.12 to 0.29, respectively, indicating modest dispersion levels but slightly higher than the 2nd round. No competency met the criteria for removal.

To address the remaining comments and ensure the framework's comprehensiveness and accuracy following the 3rd round of Delphi consultation, a virtual panel discussion was convened. This discussion involved a smaller group of Delphi experts who came together to make critical decisions and finalize the framework. The revisions stemming from the panel discussion encompassed several key aspects, including:

- Accuracy of Expressions: Improvements were made to ensure precise and clear language in the framework.
- Inclusion or Removal of Competencies: The panel confirmed whether the competencies in question should be retained or removed.
- Sequencing of Secondary Competencies: Adjustments were made to the sequence of secondary competencies for enhanced clarity and coherence.
- Feasibility Enhancement: Efforts were directed toward making competencies that were challenging to measure more feasible.
- Domain Reordering: Domains were rearranged in a logical order, following a "knowledge-skills-practice" progression.
- Language Uniformity: A consistent language style was adopted throughout the framework.

The comprehensive results of the panel discussion are summarized in Additional file 8.

### The adapted CUGH competency framework

**Fig. 2:**
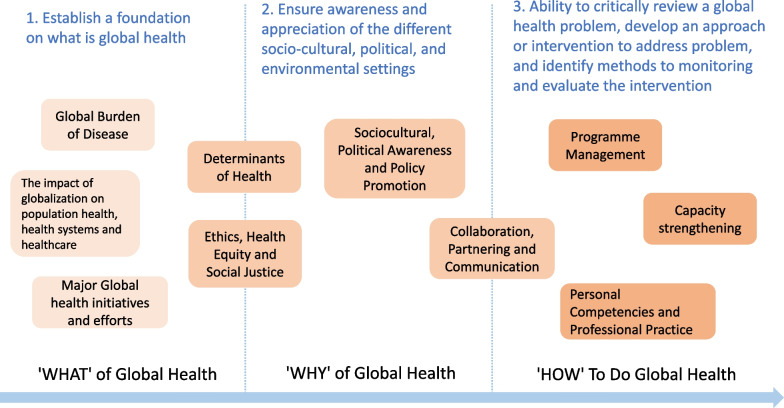
A proposed structure of the adapted CUGH global health competency framework in Chinese context

The final competency framework comprises 10 domains and 37 competencies (Table 2). To enhance clarity and comprehensibility, a structured "3W" framework for the adapted competency framework was developed (depicted in Fig. 2). This framework elucidates the interrelationships among the domains using the following components:

- *What* -This component defines the scope, elucidating what falls within the realm of global health.
- *Why* -It offers explanations regarding the significance of global health and why it is of paramount importance.
- *How* -This aspect delves into the methodologies, approaches, and techniques that can be effectively employed in the field of global health.

**Table 2:** The adapted CUGH Global health competencies in Chinese context

**1. 全球疾病负担** **Global Burden of Disease** 了解高、中、低收入国家和地区的主要疾病负担的分布及原因。 Understand the distribution and causes of major disease burden in high-, middle- and low-income countries, territories and areas
1.1了解全球主要疾病的发病和死亡原因、疾病负担指标及其变化趋势。 Understand the morbidity and mortality of major disease around the world, and the indicators and trends of disease burden
1.2能够通过获取和应用公开资料和数据了解特定人群的疾病和健康信息。 Ability to acquire disease and health information of target population through public literature and data
1.3能够分析全球卫生领域重要热点问题或挑战。 Ability to analyze key issues or challenges in the global health arena
**2. 影响健康的社会经济、环境和行为因素** **Socioeconomic, Environmental and Behavioral Determinants of Health** 了解社会、经济、环境和行为是健康的重要影响因素及其之间的相互作用, 健康不仅是没有疾病, 健康在所有相关政策中有所体现。 Understand that social, economic, environmental and behavioral factors, along with their interactions, are important determinants of health. Health is more than the absence of disease, which should be considered in all policies
2.1了解文化和宗教背景、受教育程度如何影响人们对健康和疾病的认识。 Understand how cultural context, religion and education influence perceptions of health and disease
2.2能够列出影响健康的主要社会和经济因素, 及其对医疗卫生服务可及性和质量的影响。 List major social and economic determinants of health and their effects on the access to and quality of health services
2.3知晓饮用水、食品、卫生条件、空气、土壤以及医疗设施的可及性与质量对个体和人群健康的影响。 Understand the relationship between access to and quality of water, food, sanitation, air, earth and health facilities on individual and population health
2.4能够描述影响健康的主要个体行为因素。 The ability to describe the behavioral factors of health determinants
**3. 全球化对人群健康、卫生系统和医疗服务的影响** **The Impact of Globalization on Population Health, Health Systems and Healthcare** 了解全球化如何影响人群健康、卫生系统和医疗服务。 Understand how globalization affects health, health systems and health care
3.1能够描述典型国家卫生体系类型或医疗服务模式, 及其对健康和卫生支出的影响。 Describe typical national healthcare systems or healthcare service models and their impacts on health and health care expenditure
3.2能够描述全球化进程中商贸、文化、医疗卫生等因素对本地和全球健康和医疗卫生服务/产品的影响。 Describe the impact of commerce, culture, health and other factors on local and global health care, while taking into account globalization
3.3了解知识产权制度对包括药品在内的卫生技术研发创新的激励作用和局限性。 Understand the incentives and limitations of Intellectual Property system for health technology Research & Development (R&D), including pharmaceuticals
**4. 全球卫生领域的重要倡议和行动** **Major Global Health Initiatives and Efforts** 了解全球卫生的历史和重要倡议, 能够辩证地思考全球卫生优先领域的演变以及当前的全球卫生行动。 Knowledge of global health history and major initiatives, and the ability to think critically about the changing priorities on global health issues and current global health efforts
4.1了解重要的全球卫生倡议。 Knowledge of major global health initiativies
4.2了解全球重要卫生行动。 Knowledge of major global health efforts
4.3了解全球主要疾病和卫生问题的主要干预策略。 Knowledge of major diseases around the world and major intervention strategies of public health issues
4.4了解全球卫生的历史和现状, 能够分析经验和教训。 Knowledge of global health history and its current situation, and the ability to analyze and learn from the past
**5.伦理、卫生公平和社会正义** **Ethics, Health Equity and Social Justice** 具备在运用基本伦理准则处理全球卫生问题的能力; 具备运用卫生公平和社会正义分析框架处理不同社会环境、人口学或地理学特征人群所面对的健康不公平问题的能力。 Ability to address global health issues with the basic principles of ethics; ability to address health disparities by health equity and social justice frameworks across socially, demographically, or geographically defined populations
5.1在不同经济、政治、文化和宗教背景下工作时, 或与弱势群体工作时, 能够判断卫生项目是否能够符合当地的伦理规范, 理解并能够制定出符合基本伦理准则和适合当地情境的解决方案。 Ability to identify whether health projects are in accordance with local ethics, to resolve common ethical issues and challenges that arise when working within diverse economic, political, cultural and religious contexts as well as when working with vulnerable populations
5.2具备与工作环境相关的地方和国家道德规范的意识。 Awareness of local and national codes of ethics relevant to one’s working environment
5.3能够运用国际标准中的基本原则来保护不同宗教、文化背景下的脆弱人群。 Apply the fundamental principles of international standards for the protection of human subjects in diverse cultural settings
5.4理解和认识发展中国家人口获得初级卫生保健服务的可及性和公平性的障碍。 Understand the barriers to access and equity of primary health care services for populations in developing countries
5.5能够应用卫生公平策略促使边缘和弱势群体参与影响其健康和福祉的决策。 Implement strategies to engage marginalized and vulnerable populations in making decisions that affect their health and well-being
5.6基本理解健康差异、人权和全球不公平之间的关系。 Demonstrate a basic understanding of the relationships between health disparities, human rights, and global inequities
5.7具备良好的社会责任感。 Demonstrate a commitment to social responsibility
**6.社会文化、政治意识和政策推动** **Sociocultural, Political Awareness and Policy Promotion** 社会文化和政治意识是在不同文化背景下, 在地方、区域、国家和国际政治环境中有效工作的重要前提。 Sociocultural and political awareness is the conceptual basis with which to work effectively within diverse cultural settings and across local, regional, national and international political landscapes
6.1 能够描述影响全球卫生发展的主体之间的角色及其关系, 描述全球卫生行为体的多元化、不同类别行为体在全球卫生治理中的作用、贡献和面临的挑战以及应对策略。 Describe the roles and relationships among actors that influence global health development, describe the various global health actors, the role of different types of actors in global health governance, their contribution and challenges, and coping strategies
6.2能够描述中国基本国情、中国的全球卫生角色、地位和作用, 以及新形势下中国开展全球卫生工作的主要方针和政策。 Describe China's basic national conditions, roles, and policies in global health under new situations
6.3对体制、文化、环境、社会、宗教、法律、外交、国家安全等领域的信息具有敏感性。 Awareness of the information of politics, culture, environment, society, religion, law, diplomacy and national security
6.4熟知工作国的政策程序和政治特征, 具有在复杂的政策环境下将数据、证据和方案转换成政策表述、政策文件以及将相关政策推动落实的能力。 Familiar with the policy procedures and political characteristics of the target country, with the ability to translate data, evidence and work plans into policy statements, policy documents and the implementation of relevant policies in a complex policy environment
**7. 与全球卫生相关的个人基本素养和专业实践积累** **Personal Competencies and Professional Practice** 具备自身专业或学科有关活动所需要的必备素养、知识、技能和实践经验。 The necessary competencies, knowledge, skills and practical experience needed for professional activities
7.1 能够运用工作环境的官方语言进行有效沟通, 跨文化开展工作。 Communicate effectively in the official language of the target context and the ability to work cross-culturally
7.2具备情绪管理能力、强大的心理承受能力和应对、解决冲突的技巧与能力。 Emotion management skills, strong psychological endurance and skills and abilities to cope with and resolve conflicts
7.3在专业实践的各个方面显示出诚信。 Demonstrate integrity in all aspects of professional practice
7.4具有在资源有限的环境中开展专业技术工作的能力。 Ability to apply discipline-specific skills and practice in a resource-constrained setting
**8.能力加强** **Capacity Strengthening** 能力加强是指通过分享知识、技能和资源、完善全球公共卫生项目和基础设施、促进人力资源培养, 来解决目前和未来的全球公共卫生需求。 Capacity strengthening is sharing knowledge, skills and resources for enhancing global public health programmes, infrastructure and workforce to address current and future global public health needs.
8.1能够与合作方共同评估卫生服务提供机构的卫生服务提供能力, 识别差距, 提出有针对性的建议。 Collaborate with a host or partner organization to assess the organization’s operational Capacity, identify gaps and propose corresponding recommendations
8.2能够在跨国界/跨文化的情况下, 与社区合作制定提升人员能力的策略和具体措施。 Cocreate strategies with the community to strengthen community capabilities, in a cross-border or cross cultural context
8.3能够从优化配置的角度, 向合作机构和社区提供资源整合的依据和建议。 Integrate community assets and resources to improve the health of individuals and populations
**9.合作与沟通** **Collaboration, Partnering and Communication** 合作伙伴关系是为了改善人群健康, 与各类全球卫生利益相关者开展合作, 从而推动研究、影响卫生实践和政策制定的能力, 以及与合作伙伴和团队内部建立开放式对话和有效沟通的能力。 Collaborating and partnering is the ability to select, recruit and work with a diverse range of global health stakeholders to advance research, policy and practice goals, and to foster open dialogue and effective communication with partners and within a team
9.1 具备跨学科视角和文化敏感性, 尊重和理解从事全球卫生工作的专业人士和团体所代表的独特文化、价值观、角色/职责和专业。 Exhibit interprofessional values and communication skills that demonstrate respect for, and awareness of, the unique cultures, values, roles/responsibilities and expertise represented by other professionals and groups that work in global health
9.2具有与不同文化背景的合作伙伴良好的沟通技巧和传播的能力。 Demonstrate communication skills and information dissemination skills with partners from different cultural backgrounds
9.3能够运用领导力来开展合作和提升团队效率。 Apply leadership practices that support collaborative practice and team effectiveness
**10.全球卫生项目管理** **Programme Management** 项目管理能力包括设计、实施、督导和评估全球卫生项目, 以最大程度促进全球卫生政策的可及性、有效性、可持续地改善卫生服务、促进健康。 Programme management is ability to design, implement, supervise and evaluate global health programmes to maximize contributions to effective policy, enhanced practice, and improved and sustainable health outcomes
10.1具备项目设计能力, 能够与当地人员共同基于循证原则对当地人群健康需求进行评估和策略分析。 Plan project, collaborate with local personnel, to analyze the health needs of target populations by evidence-based principles
10.2具备组织项目实施能力, 能够应用项目管理技能, 因地制宜实施项目或开展干预措施。 Implement project, apply project management skills, implement interventions according to local conditions
10.3具备项目督导评估能力, 促进项目可持续发展。 Supervise and evaluate project to promote sustainable development of the project

A full version of Table 2 with footnotes is available in Additional file 9

### Basic information of the priority survey participants

Out of the 72 experts contacted for the priority survey, a total of 51 responded. These participants encompassed a diverse group, consisting of 38 out of the 48 Delphi experts, 4 out of the 9 additional Chinese experts, and 9 out of the 15 additional international experts.

For the Chinese experts specifically, 78.57% (33 out of 42) held senior or sub-senior positions, while 71.43% (30 out of 42) were employed in the public health sector or universities. An impressive 83.33% (35 out of 42) boasted more than 6 years of experience in the field of global health.

In contrast, all of the international experts held senior-level positions and possessed over 10 years of work experience in global health. Among them, 66.67% (6 out of 9) were affiliated with universities or the public health sector.

### Prioritized competencies for training Chinese public health professionals

**Fig. 3:**
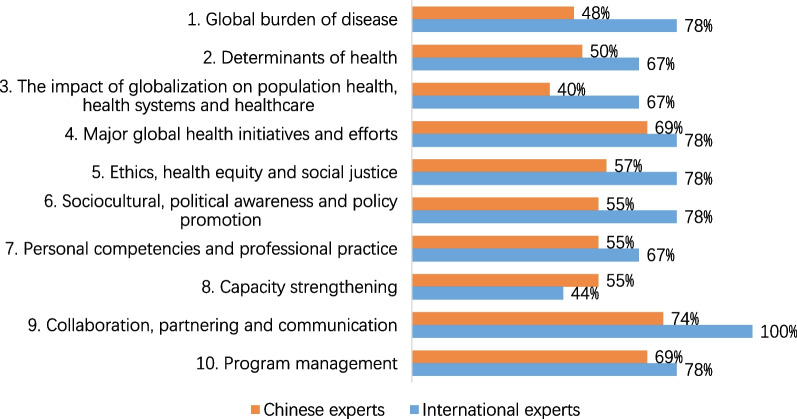
Prioritized competencies for Chinese public health professionals

The competency that emerged as the highest priority for training Chinese public health professionals, as agreed upon by the majority of both Chinese and international experts, is “Collaboration, Partnering, and Communication”. This competency received resounding support, with 74% (31 out of 42) of Chinese experts and 100% (9 out of 9) of international experts endorsing it (as depicted in Fig. 3). Following closely in priority are "Programme Management" and "Major Global Health Initiatives and Efforts," with 69% (29 out of 42) of Chinese experts and 78% (7 out of 9) of international experts emphasizing their importance.

The rationale behind prioritizing this competency was consistent among Chinese and international experts. It hinged on addressing the distinct challenges and specific needs or demands within the realm of Chinese public health professionals. Importantly, it excluded areas that could be adequately covered through short-term training programs. Notably, international experts highlighted the critical importance of “context” in their reasoning. They emphasized that Chinese global health professionals must possess the capacity to "understand different contexts regarding the burden of disease and demography," "address challenges related to the implementation of public health initiatives in diverse contexts," "cultivate an awareness and appreciation of various socio-cultural, political, and environmental settings," and "build capacities to operate effectively in non-Chinese contexts." Furthermore, they emphasized the “practical issues”. For example, an expert noted that Chinese public health professionals are already well-versed in health-related knowledge, and the focus should shift toward practical aspects—specifically, the ability to critically analyze public health problems, develop intervention strategies, and establish methods for monitoring and evaluating interventions.

### Recommendations from the priority survey participants

In the recommendation section of the priority survey, both Chinese and international experts underscored the following key points for individuals and institutions to improve their global health capacities:

1. Learning from practice and exposure to different contexts: Experts emphasized the value of practical learning and gaining exposure to diverse contexts. They suggested that this could be achieved through internships, exchange programs with collaborative institutions, or engaging in fieldwork.
2. Learning from case studies: Case studies were identified as a valuable learning tool. Experts recommended comparing and contrasting case studies from Western China and other regions or drawing insights from major studies conducted by organizations such as the Organisation for Economic Co-operation and Development's Development Assistance Committee (OECD/DAC), the World Bank, and bilateral agencies, which provide insights from development projects.
3. Learning from group discussions: Chinese experts highlighted the significance of group discussions as a primary training approach. This collaborative learning method fosters dialogue and shared insights among professionals.
4. Promoting self-reflection, active learning, critical thinking, and open communication: International experts stressed the importance of nurturing self-reflection, active learning, critical thinking, and open communication among individuals seeking to excel in global health. These qualities are seen as essential for adaptability in new situations and fostering effective global working relationships.
5. Institutional capacity strengthening and partnership building: Recommendations were made for institutions to strengthen their institutional capacity and actively engage in partnerships with other institutions. Additionally, it was suggested that institutions should identify specific areas within the field of global health where China can make a meaningful impact, thereby informing long-term strategic planning.

## Discussion

In the global context, the development of global health competencies has been a dynamic area of research and practice since the 2000s. Plenty of studies have emerged, employing various methodologies such as literature reviews, interviews, surveys, and the review of academic programs tailored for medical, nursing, or global health students [19–29]. Notably, a substantial portion of these initiatives originated from high-income countries like the United States, the United Kingdom, Canada, and Australia, where the concept of global health first took root. However, it is worth highlighting the predominance of this trend and the associated geographic bias towards high-income countries. Recognizing this disparity, some researchers have called for the validation and adaptation of these competencies in Asian countries [30]. In China, efforts to introduce global health education began with the proposal of course modules for undergraduate programs, marking the launch of global health programs in universities as early as 2011 [31–33]. Despite these commendable initiatives, a critical gap persisted—the absence of a competency framework designed specifically for professionals already established in their careers who aspire to gain global health experience. As China continues to play an increasingly prominent role in global health initiatives, the adapted competency framework serves as a valuable resource, aligning training programs with the specific needs and challenges encountered by Chinese professionals engaged in global health endeavors.

### What’s new in this framework

Comparing with the original CUGH framework (see Additional file 10 for comparison), the adapted framework introduces several adjustments. These changes include:

- Enhanced analytical proficiency: Addition of competencies related to analyzing key global health issues, understanding intellectual property systems in health technology development, and applying historical insights to contemporary global health challenges (competency 1.3, 3.3, 4.4).
- Expanded global health governance and policy acumen: Enrichment of competencies covering roles and relationships among global health actors, China's roles and policies, political awareness, and the ability to navigate complex policy environments (competency 6.1, 6.2, 6.3, 6.4).
- Improved effective communication and emotional intelligence: Inclusion of competencies emphasizing effective communication skills, cross-cultural competence, emotion management, psychological endurance, and conflict resolution abilities (competency 7.1, 7.2).

The Chinese Delphi panel largely agrees with the CUGH framework, highlighting its well-developed nature. However, this adapted framework offers distinct value by adapting the competencies to a different setting, reducing potential biases that may arise from the original development process, which primarily involved US-based panelists. This adaptation recognizes the unique needs and challenges faced by the Chinese workforce in global health. For instance, the inclusion of competency 7.1, which focuses on effective communication in the official language of the target context, addresses a language barrier that may not be as relevant for a US-based workforce. Additionally, competencies in domain 6 underscore the importance of understanding the political, social, cultural, and policy aspects of both China and recipient countries. These competencies empower the workforce to navigate diverse global contexts and contribute effectively.

### Core debates among the Delphi experts

Throughout the Delphi process, several key debates emerged among the experts, reflecting important considerations in the development of competency frameworks for global health professionals:

One central debate revolved around the feasibility of individuals possessing all the competencies outlined in the framework. Some experts raised the concern that it might be unrealistic for a single professional to possess every competency listed. The recognition that not all competencies will be equally relevant to every individual or program led to reflections on the current status of China's public health professionals. It also highlighted the need for further analysis to tailor the competencies for different levels of professionals and those with diverse scientific backgrounds. This debate underscores the importance of adaptability and context specificity in competency development.

Another significant debate revolved around how to effectively assess the competencies outlined in the framework. Assessing competencies has long been a challenge in competency-based education [34]. The experts reached a consensus that qualitative methods could be employed, despite the inherent difficulties. This includes approaches such as self-assessment, allowing learners to evaluate their own work after receiving proper guidance to enhance the validity and reliability of assessments. Additionally, involving a third party, such as a co-worker working alongside the learner, was suggested as a means to assess competencies [35]. These discussions reflect the ongoing exploration of innovative assessment methods in competency-based education.

The experts also grappled with the challenge of avoiding overlaps among competencies. They recognized that certain overlaps were inevitable, as each competency may have specific emphases under different domains. However, to enhance clarity and coherence within the framework, efforts were made to minimize overlaps. This was achieved by merging closely related competencies into broader domains that could encompass both competencies. This debate highlights the need for precision and conciseness in competency frameworks while acknowledging the interconnected nature of competencies in practice.

### Bottlenecks of China’s public health professionals

China's public health professionals face several bottlenecks and challenges, which have been highlighted through the Delphi consultation process and other research. One major bottleneck is lacking of international experience and the limited exposure of Chinese global health workers to foreign cultures. This lack of exposure makes it challenging for these professionals to fully adapt, both physically and intellectually, to the localities where they work. The global nature of health work often requires individuals to operate effectively in diverse and unfamiliar cultural contexts. Without adequate exposure and cross-cultural training, Chinese public health professionals may struggle to navigate these challenges effectively. Language barriers and communication gaps with international co-workers are another obstacle associate with the lacking of international experience. Effective communication is essential in global health collaboration, as it facilitates the exchange of knowledge, ideas, and best practices. When language becomes a barrier, it can impede teamwork, hinder information sharing, and limit the impact of global health initiatives. Similar challenges have been reported among China's Overseas Medical Teams, which have been providing health assistance in African countries for over 60 years [36]. These medical teams have encountered language barriers and difficulties adapting to local contexts [37]. These challenges can impact the effectiveness of medical missions and require strategies for mitigation.

### Suggested steps for institutions and individuals intend to use the framework

For institutions and individuals intending to use the framework, here are recommended steps to integrate and apply the competencies:

For Institutions: (1) Begin by reviewing the institution's existing curricula in the context of the competency framework. Identify areas where the current curriculum aligns with the competencies and where there are gaps. (2) Re-design training modules to ensure that the competency priorities are adequately addressed within the curriculum. Adjust the distribution of time and resources to reflect the importance of each competency. (3) Map out the available teaching, learning, and field practice resources. Consider introducing e-learning and online resources, promote interdisciplinary co-teaching [38], and establish connections with international institutions to share educational materials and resources [39]. (4) Engage faculty members in harmonizing the resources and reconfirming the study objectives and outcomes of each module. Ensure that the faculty is aligned with the competency-based approach. (5) Innovate teaching methods by introducing interactive activities and novel teaching approaches that encourage critical thinking and open communication. Examples include case-based studies, group discussions, thematic seminars, and flipped classroom techniques. (6) Offer field practice opportunities to expose trainees to international contexts. Practical experience in global health settings can enhance the application of competencies in real-world scenarios. (7) Continuously test, evaluate, and modify the training program to ensure that it effectively aligns with the competency framework. Gather feedback from students, faculty, and experts to make necessary improvements.

For Individuals: (1) Gain hands-on experience by working in developing or low-income countries. Practical experience is invaluable for understanding global health challenges and solutions. (2) Develop critical thinking skills by analyzing global health problems and potential solutions. Consider the effectiveness of interventions and explore alternative approaches. (3) Identify and gather data from internationally available sources, such as the World Health Organization. Utilize global health datasets to inform the understanding of health patterns and determinants. (4) Characterize local, regional, and global patterns of health and disease, focusing on socio-cultural and environmental determinants. Understand the complex interplay of factors influencing health outcomes. (5) Develop appropriate global health interventions and evaluate their impact. This process will help individuals become familiar with major global health initiatives and enhance the capacity to analyze and interpret information for policy and program decision-making.

This study has several limitations. First, the study focused exclusively on public health professionals engaged in global health activities in China. Medical workers involved in global missions were not included in the targeted population. This limitation may restrict the applicability of the framework to a broader range of healthcare professionals. Second, the accuracy of the English-to-Chinese translation of the competencies may impact how well the experts comprehend the framework. Variations in translation can introduce subtle differences in interpretation, potentially affecting the results and applicability of the competencies.

## Conclusions

This study has adapted the CUGH framework for global health competencies in the Chinese context. The revised framework offers a useful tool for self-assessment, training, and job description development among Chinese public health professionals engaged in global health activities. In addition, the study identified three key priorities for capacity building among China's public health professionals, which include "Collaboration, Partnering and Communication", "Programme Management," and "Major Global Health Initiatives and Efforts." These priorities reflect the specific needs and challenges faced by Chinese professionals in the global health arena. To improve global health capacity in China, individuals and institutions are encouraged to promote active learning, critical thinking, and open communication. Learning through practical experience and case studies can enhance professionals' understanding of global health contexts. Strengthening institutional capacity, fostering partnerships, and identifying China's unique role in global health are also essential recommendations. Moving forward, it is important to further define each competency, integrate them into the existing educational structure, develop suitable assessment instruments, and rigorously test the curriculum. This iterative process will ensure the continuous improvement and effectiveness of the framework in enhancing China's global health capabilities.

### Supplementary Information

**Additional file 1:** The Delphi questionnaire for validating the CUGH competency framework in Chinese context (in Chinese).

**Additional file 2**: The questionnaire for the priority survey (in Chinese).

**Additional file 3**: The questionnaire for the priority survey (in English).

**Additional file 4**: Values of the Delphi experts’ judgment criterion (Ca) and familiarity (Cs) to each competency.

**Additional file 5**: The characteristics of the Delphi experts and the response rates.

**Additional file 6**: Experts’ scores and revisions of 1st round Delphi consultation.

**Additional file 7**: Experts’ scores and revisions of 2nd round Delphi consultation.

**Additional file 8**: Experts’ scores of 3rd round Delphi consultation and results of the panel discussion.

**Additional file 9**: Full version of the adapted CUGH global health competencies in Chinese context.

**Additional file 10**: Comparison of the CUGH framework and the adapted CUGH framework in Chinese setting.

## Data Availability

The data contained in this manuscript are available upon a reasonable request to the corresponding author.
