# Supplementary material for: Adaptation of the CUGH global health competency framework in the Chinese context: a mixed-methods study
Source: Glob Health Res Policy. 2023 Nov 2;8:46. doi: 10.1186/s41256-023-00327-w (PMC10621075; doi:10.1186/s41256-023-00327-w)
Supplement: Supplementary file 1 — Additional file 1: The Delphi questionnaire for validating the CUGH competency framework in Chinese context (in Chinese). [file 41256_2023_327_MOESM1_ESM.docx]

**Additional file 1. The Delphi questionnaire for validating the CUGH competency framework in Chinese context (in Chinese)**

Below is the 1^st^ round Delphi consultation questionnaire including: (1) study introduction, (2) informed consent form, (3) instructions for completing the questionnaire, (4) the main questionnaire with information for evaluating experts’ authority, and (5) experts’ profile.

**中国公共卫生人员全球卫生素养评价指标的构建**

**专家咨询说明**

尊敬的专家：

您好！

全球卫生是以公共卫生的理论为基础，利用不同学科的知识与技术理解与改善全球人口的健康的科学。素养是一种综合能力，由基于个人教育实践所形成的知识、技能、经验、价值观等发展而来。随着中国越来越多的公共卫生人员加入全球卫生事业，对中国参与全球卫生人员的素养提出了更高要求。但是，关于我国公共卫生人员从事全球卫生应该具备哪些主要素养、如何提高相关素养的研究尚不多见。美国全球健康大学联盟（Consortium of Universities for Global Health，简称CUGH）在2015年建立了全球卫生素养评价体系（Competency Model），据此开展全球卫生人员的培训、招聘，培养了一批适应全球多文化工作背景、通晓全球卫生知识和技能的全球卫生人员，推动了全球卫生人才队伍的建设。

本研究拟以CUGH的全球卫生素养评价体系为模版，通过多轮专家征询意见，以构建我国公共卫生人员从事全球卫生所需具备的素养评价指标；同时，分析我国全球卫生人员相对缺乏或亟待提升的素养，为培养高素质的全球卫生人才提供决策依据。

为此，我们邀请您根据您的实践、经验与理解，在CUGH体系的基础上，对建立我国公共卫生人员全球卫生素养评价指标的重要性和可行性进行评价，并对相应指标提出修改建议。您的判断和意见将是推动我国全球卫生人才队伍建设的重要依据，对“一带一路”倡议在卫生领域的实施具有重要意义。

衷心感谢您对我们工作的支持！

**中国疾病预防控制中心寄生虫病预防控制所全球卫生中心**

2018年8月24日

**知情同意书**

**研究目的：**构建中国公共卫生人员从事全球卫生所需要具备的素养评价指标；提出提升中国全球卫生人员素养的对策。

**研究方法：**本研究采用专家咨询法（德尔菲法）。您将在未来的2个月中，以书面或邮件形式参与至少3次调查，每次完成调查需要20-30分钟。

**专家的选择：**您的经验有助于构建中国公共卫生人员全球卫生素养评价指标体系，因此特邀请您参与这项研究。

**自愿参与：**您参与这项研究是完全自愿的。

**结果分享:**本研究所得到的结果将与每位参与专家分享。

**退出研究:**如您不希望继续参与本研究，您可以随时通知我们退出本研究。

我被邀请参加“中国公共卫生人员全球卫生素养评价指标的建立”这一研究。

我已经读过前面的信息，本人同意自愿参与本研究。

参与专家姓名（印刷体）_______

参与专家签名_________________（**如不便添加签名，可邮件回复“同意参加”**）

日期_________________________（年月日）

**中国公共卫生人员全球卫生素养评价指标**

**专家咨询表（第1轮）**

**填表说明**

**一、**本研究的目的是构建中国公共卫生人员从事全球卫生所需具备的素养评价指标，提出推动中国全球卫生人员素养的对策。

二、本轮为第一轮专家咨询，期望能汇集全球卫生领域专家智慧，建立**“中国公共卫生人员全球卫生素养”评价指标**。首轮指标参考了美国全球健康大学联盟（简称CUGH）全球卫生素养评价体系。

三、请根据以下程序完成本咨询表填写：

（一）请对照“指标内容”，从以下两个方面综合评价表中所列条目，进行评分。两个方面包括：

- 重要性：指该指标在代表全球卫生素养内容方面的重要程度。
- 可行性：指该指标在多大程度上能够客观地被评价和获得。

请对各指标的上述两个方面给出您的评分， 1表示“很不重要/很不可行”，2表示“较不重要/较不可行”，3表示“一般”，4表示“比较重要/比较可行”，5表示“很重要/很可行”。

（二）填写您给出各项指标判断依据的依赖程度（Ca）。

您在对每一项指标进行评分判断时，可能会依赖相应的依据，如：理论依据、实践经验、对国内外同行的了解和直觉判断等。请您在对某项指标进行评分时，就以上四种依据选择相应的依赖程度（高、中、低）。

（三）填写您对各项指标的熟悉程度系数（Cs），请选择以下分值填写：

| 指标 | 熟悉程度 | 熟悉程度最高 | 很熟悉 | 较熟悉 | 一般 | 不熟悉 | 很不熟悉 |
| --- | --- | --- | --- | --- | --- | --- | --- |
| X | 系 数 | 1.0 | 0.8 | 0.6 | 0.4 | 0.2 | 0 |

如对某项指标的熟悉程度介于两级别之间，可以考虑评分为0.9、0.7、0.5、0.3、0.1。

（四）填写示例

如果说明仍不够明确，请按照下列示例填写：

| **指标内容** | **重要性**  **（1-5）** | **可行性（1-5）** | **对该项指标判断依据的依赖程度（Ca）**  **（高、中、低）** | | | | **对该项指标熟悉程度系数（Cs）**  **（1.0-0）** | **对该项指标的修改建议** |
| --- | --- | --- | --- | --- | --- | --- | --- | --- |
|  |  |  | **理论依据** | **实践经验** | **国内外同行的了解** | **直觉** |  |  |
| **X** | 4 | 3 | 高 | 中 | 低 | 中 | 0.8 |  |

示例说明：评分者对指标X的重要性和可行性做出的评分分别为4分和3分。在进行该指标相关性评分时，对自己“理论依据”的依赖程度高，对“实践经验”和 “靠直觉判断”的依赖程度为中等，对“国内外同行的了解”依赖程度较低。评分者对指标X很熟悉，相应Cs为0.8。对指标X没有建议。

**三、注意事项**

（一）在对各项指标进行评分时，请务必遵循层次原则，即考虑二级指标对一级指标的贡献程度。

（二）如对指标有其他建议、意见或删减，请在修改意见栏中说明；如有增加一级指标，请在表12中填写：指标名称、指标描述和增加理由；如有增加二级指标，请在表1-表11中分别填写：指标描述和增加理由。

（三）附上CUGH全球卫生素养指标供您参考。

（四）请务必填写完整，不要留空项。

（五）请专家妥善保存本轮咨询表，以便下一轮咨询时参考。

（六）在咨询表最后，请务必填写好您的个人资料及关键信息，以便于我们和您保持联系。

本次为第一轮德尔菲专家咨询，恳请您在**9月2日前**尽快填写好本咨询表，用电子邮件返回。返回邮箱：[dingwei@nipd.chinacdc.cn](mailto:dingwei@nipd.chinacdc.cn) 。谢谢！

联系人：丁玮、官亚宜

联系电话：13681967703、13818987832

**第一轮德尔菲专家咨询表（表1至13）**

**表1：对指标1的评价**

| **指标**  **序号** | **指标层次** | **指标内容** | **重要性**  **（1-5）** | **可行性（1-5）** | **对该指标判断依据的依赖程度(Ca)**  （高，中，低） | | | | **对该指标的熟悉程度系数(Cs)**  （1.0-0，1.0=熟悉程度最高，0=很不熟悉） | **对该指标的修改建议** |
| --- | --- | --- | --- | --- | --- | --- | --- | --- | --- | --- |
|  |  |  |  |  | **理论依据** | **实践经验** | **国内外同行的了解** | **直觉** |  |  |
| **1** | 一级 | **1. 了解全球疾病负担**  基本了解高、中、低收入地区之间发病和死亡的主要原因及差异，了解全球卫生领域的重要倡议和行动。 |  |  |  |  |  |  |  |  |
|  | 二级 | 1.1 能够描述全球发病和死亡的主因、疾病风险如何因地而异。 |  |  |  |  |  |  |  |  |
|  |  | 1.2能够描述全球重要公共卫生倡议和行动。  例如：联合国千年发展目标、抗击艾滋病、结核病和疟疾全球基金。 |  |  |  |  |  |  |  |  |
|  |  | 1.3能够获取公开数据，了解人群健康状况。  例如：公共卫生监测数据、重要统计数据、注册表、调查数据、电子健康记录、卫生规划数据等。 |  |  |  |  |  |  |  |  |
|  |  | **如您认为有其他应当纳入的二级指标，请填写在此**（行数可自行增加）：  指标描述：  增加理由： | | | | | | | | |

**表2：对指标2的评价**

| **指标**  **序号** | **指标层次** | **指标内容** | **重要性**  **（1-5）** | **可行性（1-5）** | **对该指标判断依据的依赖程度(Ca)**  （高，中，低） | | | | **对该指标的熟悉程度系数(Cs)**  （1.0-0，1.0=熟悉程度最高，0=很不熟悉） | **对该指标的修改建议** |
| --- | --- | --- | --- | --- | --- | --- | --- | --- | --- | --- |
|  |  |  |  |  | **理论依据** | **实践经验** | **国内外同行的了解** | **直觉** |  |  |
| **2** | 一级 | **2. 理解健康和卫生服务的全球化**  了解全球化如何影响健康、卫生系统和医疗服务。 |  |  |  |  |  |  |  |  |
|  | 二级 | 2.1能够描述不同国家的卫生体系或医疗服务模式，以及它们对各国的健康和对卫生保健支出的影响。 |  |  |  |  |  |  |  |  |
|  |  | 2.2能够描述全球化的卫生实践、商业、文化、国际协议和国际组织对本地和全球的健康和医疗卫生的质量和可及性的影响。 |  |  |  |  |  |  |  |  |
|  |  | 2.3能够描述旅游和贸易如何影响传染病和慢性病的传播。 |  |  |  |  |  |  |  |  |
|  |  | 2.4能够描述全球卫生人力资源可及性和流动性的总体趋势和影响因素。 |  |  |  |  |  |  |  |  |
|  |  | **如您认为有其他应当纳入的二级指标，请填写在此处**（行数可自行增加）：  指标描述：  增加理由： | | | | | | | | |

**表3：对指标3的评价**

| **指标**  **序号** | **指标层次** | **指标内容** | **重要性**  **（1-5）** | **可行性（1-5）** | **对该指标判断依据的依赖程度(Ca)**  （高，中，低） | | | | **对该指标的熟悉程度系数(Cs)**  （1.0-0，1.0=熟悉程度最高，0=很不熟悉） | **对该指标的修改建议** |
| --- | --- | --- | --- | --- | --- | --- | --- | --- | --- | --- |
|  |  |  |  |  | **理论依据** | **实践经验** | **国内外同行的了解** | **直觉** |  |  |
| **3** | 一级 | **3.了解健康的影响因素（社会和环境）**  了解社会、经济和环境因素是健康的重要决定因素，健康不仅是没有疾病。 |  |  |  |  |  |  |  |  |
|  | 二级 | 3.1 能够描述文化背景如何影响人们对健康和疾病的认识。 |  |  |  |  |  |  |  |  |
|  |  | 3.2 能够列出影响健康的主要社会和经济决定因素、这些因素对卫生服务可及性和质量的影响和对不同国家之间和同一国家不同地区之间的发病和死亡差异的影响。 |  |  |  |  |  |  |  |  |
|  |  | 3.3能够描述饮用水、卫生设施、食品和空气的可及性和质量与个体和人群健康之间的关系。 |  |  |  |  |  |  |  |  |
|  |  | **如您认为有其他应当纳入的二级指标，请填写在此处**（行数可自行增加）：  指标描述：  增加理由： | | | | | | | | |

**表4：对指标4的评价**

| **指标**  **序号** | **指标层次** | **指标内容** | **重要性**  **（1-5）** | **可行性（1-5）** | **对该指标判断依据的依赖程度(Ca)**  （高，中，低） | | | | **对该指标的熟悉程度系数(Cs)**  （1.0-0，1.0=熟悉程度最高，0=很不熟悉） | **对该指标的修改建议** |
| --- | --- | --- | --- | --- | --- | --- | --- | --- | --- | --- |
|  |  |  |  |  | **理论依据** | **实践经验** | **国内外同行的了解** | **直觉** |  |  |
| **4** | 一级 | **4.能够开展能力提升活动**  能力提升是指为了满足当前和未来的全球公共卫生需求，通过分享知识、技能和资源，提升全球公共卫生项目、基础设施和人力资源。 |  |  |  |  |  |  |  |  |
|  | 二级 | 4.1 能够为机构评估其运作能力。 |  |  |  |  |  |  |  |  |
|  |  | 4.2 能够为社区制定相关策略，提高社区能力，减少卫生不公平，促进社区健康。 |  |  |  |  |  |  |  |  |
|  |  | 4.3 能够整合社区各类资产和资源，改善个体和人群的健康状况。 |  |  |  |  |  |  |  |  |
|  |  | 4.4 能够发现保障项目可持续性的途径。 |  |  |  |  |  |  |  |  |
|  |  | **如您认为有其他应当纳入的二级指标，请填写在此处**（行数可自行增加）：  指标描述：  增加理由： | | | | | | | | |

**表5：对指标5的评价**

| **指标**  **序号** | **指标层次** | **指标内容** | **重要性**  **（1-5）** | **可行性（1-5）** | **对该指标判断依据的依赖程度(Ca)**  （高，中，低） | | | | **对该指标的熟悉程度系数(Cs)**  （1.0-0，1.0=熟悉程度最高，0=很不熟悉） | **对该指标的修改建议** |
| --- | --- | --- | --- | --- | --- | --- | --- | --- | --- | --- |
|  |  |  |  |  | **理论依据** | **实践经验** | **国内外同行的了解** | **直觉** |  |  |
| **5** | 一级 | **5.合作与沟通能力**  合作伙伴关系是指选择、招聘各类全球卫生利益相关者并开展合作，从而推动研究、政策制定和目标实现的能力，以及与合作伙伴和团队内部建立开放式对话和有效沟通的能力。 |  |  |  |  |  |  |  |  |
|  | 二级 | 5.1 能够动员多元的社区合作伙伴代表，并促进相互学习。 |  |  |  |  |  |  |  |  |
|  |  | 5.2. 能够运用外交技巧，与社区合作伙伴建立信任关系。 |  |  |  |  |  |  |  |  |
|  |  | 5.3能够与社区合作伙伴和全球合作伙伴交流经验教训。 |  |  |  |  |  |  |  |  |
|  |  | 5.4 具备跨专业视角和沟通技巧，能够对全球卫生工作的专业人士和团体所代表的独特文化、价值观、角色/职责和专业显示出尊重和理解。 |  |  |  |  |  |  |  |  |
|  |  | 5.5 能够认识到个体在技术、知识和能力方面的局限性。 |  |  |  |  |  |  |  |  |
|  |  | 5.6 能够运用领导力来支持合作实践和提升团队效率。 |  |  |  |  |  |  |  |  |
|  |  | **如您认为有其他应当纳入的二级指标，请填写在此处**（行数可自行增加）：  指标描述：  增加理由： | | | | | | | | |

**表6：对指标6的评价**

| **指标**  **序号** | **指标层次** | **指标内容** | **重要性**  **（1-5）** | **可行性（1-5）** | **对该指标判断依据的依赖程度(Ca)**  （高，中，低） | | | | **对该指标的熟悉程度系数(Cs)**  （1.0-0，1.0=熟悉程度最高，0=很不熟悉） | **对该指标的修改建议** |
| --- | --- | --- | --- | --- | --- | --- | --- | --- | --- | --- |
|  |  |  |  |  | **理论依据** | **实践经验** | **国内外同行的了解** | **直觉** |  |  |
| **6** | 一级 | **6.伦理道德理解**  具备全球卫生背景下处理问题的基本道德准则。 |  |  |  |  |  |  |  |  |
|  | 二级 | 6.1在不同经济、政治和文化背景下工作时，或在资源匮乏环境中与弱势群体工作时，能够显示出理解并有能力解决全球健康问题中出现的常见的伦理问题和挑战。 |  |  |  |  |  |  |  |  |
|  |  | 6.2具备与工作环境相关的地方和国家道德规范的意识。 |  |  |  |  |  |  |  |  |
|  |  | 6.3运用国际标准的基本原则来保护不同文化背景下的人群。 |  |  |  |  |  |  |  |  |
|  |  | **如您认为有其他应当纳入的二级指标，请填写在此处**（行数可自行增加）：  指标描述：  增加理由： | | | | | | | | |

**表7：对指标7的评价**

| **指标**  **序号** | **指标层次** | **指标内容** | **重要性**  **（1-5）** | **可行性（1-5）** | **对该指标判断依据的依赖程度(Ca)**  （高，中，低） | | | | **对该指标的熟悉程度系数(Cs)**  （1.0-0，1.0=熟悉程度最高，0=很不熟悉） | **对该指标的修改建议** |
| --- | --- | --- | --- | --- | --- | --- | --- | --- | --- | --- |
|  |  |  |  |  | **理论依据** | **实践经验** | **国内外同行的了解** | **直觉** |  |  |
| **7** | 一级 | **7.专业实践积累**  具备专业或学科有关活动所需要的必备知识和技能。 |  |  |  |  |  |  |  |  |
|  | 二级 | 7.1在专业实践的各个方面展示诚信和对他人的尊重。 |  |  |  |  |  |  |  |  |
|  |  | 7.2能够发现并阐明在资源缺乏环境下所存在的健康和卫生服务障碍，包括当地和国际影响因素。 |  |  |  |  |  |  |  |  |
|  |  | 7.3在资源有限的环境中，能够实践临床或专业技术能力。 |  |  |  |  |  |  |  |  |
|  |  | **如您认为有其他应当纳入的二级指标，请填写在此处**（行数可自行增加）：  指标描述：  增加理由： | | | | | | | | |

**表8：对指标8的评价**

| **指标**  **序号** | **指标层次** | **指标内容** | **重要性**  **（1-5）** | **可行性（1-5）** | **对该指标判断依据的依赖程度(Ca)**  （高，中，低） | | | | **对该指标的熟悉程度系数(Cs)**  （1.0-0，1.0=熟悉程度最高，0=很不熟悉） | **对该指标的修改建议** |
| --- | --- | --- | --- | --- | --- | --- | --- | --- | --- | --- |
|  |  |  |  |  | **理论依据** | **实践经验** | **国内外同行的了解** | **直觉** |  |  |
| **8** | 一级 | **8. 理解卫生公平和社会正义**  卫生公平和社会正义是为解决不同社会环境、人口学或地理学特征人群的健康不公平的策略分析框架。 |  |  |  |  |  |  |  |  |
|  | 二级 | 8.1能够在解决全球卫生问题时，运用社会正义和人权原则。 |  |  |  |  |  |  |  |  |
|  |  | 8.2能够应用策略使边缘和弱势群体参与影响其健康和福祉的决策。 |  |  |  |  |  |  |  |  |
|  |  | 8.3基本理解健康、人权和全球不公平之间的关系。 |  |  |  |  |  |  |  |  |
|  |  | 8.4能够描述世界卫生组织在联结健康与人权中所起的作用，能够描述《世界人权宣言》和《人体生物医学研究国际伦理指南》。 |  |  |  |  |  |  |  |  |
|  |  | 8.5 具备社会责任感。 |  |  |  |  |  |  |  |  |
|  |  | 8.6理解和认识发展中国家卫生人力资源危机以及导致这种危机的因素和解决策略。 |  |  |  |  |  |  |  |  |
|  |  | **如您认为有其他应当纳入的二级指标，请填写在此处**（行数可自行增加）：  指标描述：  增加理由： | | | | | | | | |

**表9：对指标9的评价**

| **指标**  **序号** | **指标层次** | **指标内容** | **重要性**  **（1-5）** | **可行性（1-5）** | **对该指标判断依据的依赖程度(Ca)**  （高，中，低） | | | | **对该指标的熟悉程度系数(Cs)**  （1.0-0，1.0=熟悉程度最高，0=很不熟悉） | **对该指标的修改建议** |
| --- | --- | --- | --- | --- | --- | --- | --- | --- | --- | --- |
|  |  |  |  |  | **理论依据** | **实践经验** | **国内外同行的了解** | **直觉** |  |  |
| **9** | 一级 | **9.具备项目实施与管理能力**  项目管理能力包括设计、实施和评估全球卫生项目，以最大程度促进政策的有效性、促进全球卫生实践、可持续地改善健康。 |  |  |  |  |  |  |  |  |
|  | 二级 | 9.1 能够设计、实施和评估基于证据的项目。 |  |  |  |  |  |  |  |  |
|  |  | 9.2 能够在项目规划、实施和评估中，应用项目管理技能。 |  |  |  |  |  |  |  |  |
|  |  | **如您认为有其他应当纳入的二级指标，请填写在此处**（行数可自行增加）：  指标描述：  增加理由： | | | | | | | | |

**表10：对指标10的评价**

| **指标**  **序号** | **指标层次** | **指标内容** | **重要性**  **（1-5）** | **可行性（1-5）** | **对该指标判断依据的依赖程度(Ca)**  （高，中，低） | | | | **对该指标的熟悉程度系数(Cs)**  （1.0-0，1.0=熟悉程度最高，0=很不熟悉） | **对该指标的修改建议** |
| --- | --- | --- | --- | --- | --- | --- | --- | --- | --- | --- |
|  |  |  |  |  | **理论依据** | **实践经验** | **国内外同行的了解** | **直觉** |  |  |
| **10** | 一级 | **10. 具备社会文化和政治意识**  社会文化和政治意识是指在不同文化背景下，在地方、区域、国家和国际政治环境中有效工作的框架基础。 |  |  |  |  |  |  |  |  |
|  | 二级 | 10.1 能够描述影响全球卫生发展的主体之间的角色及其关系。 |  |  |  |  |  |  |  |  |
|  |  | **如您认为有其他应当纳入的二级指标，请填写在此处**（行数可自行增加）：  指标描述：  增加理由： | | | | | | | | |

**表11：对指标11的评价**

| **指标**  **序号** | **指标层次** | **指标内容** | **重要性**  **（1-5）** | **可行性（1-5）** | **对该指标判断依据的依赖程度(Ca)**  （高，中，低） | | | | **对该指标的熟悉程度系数(Cs)**  （1.0-0，1.0=熟悉程度最高，0=很不熟悉） | **对该指标的修改建议** |
| --- | --- | --- | --- | --- | --- | --- | --- | --- | --- | --- |
|  |  |  |  |  | **理论依据** | **实践经验** | **国内外同行的了解** | **直觉** |  |  |
| **11** | 一级 | **11.战略分析能力**  “战略分析是利用系统思维来分析影响健康趋势形成的各种复杂关联因素，从而在地方、国家和国际层面设计项目。 |  |  |  |  |  |  |  |  |
|  | 二级 | 11.1能够发现人口特征和其他主要因素如何影响特定人群的发病、死亡和失能的模式。 |  |  |  |  |  |  |  |  |
|  |  | 11.2能够开展社区健康需求评估。 |  |  |  |  |  |  |  |  |
|  |  | 11.3能够开展不同文化、经济和卫生背景下的形势分析。 |  |  |  |  |  |  |  |  |
|  |  | 11.4能够根据形势分析结果设计针对特定环境的健康干预措施。 |  |  |  |  |  |  |  |  |
|  |  | **如您认为有其他应当纳入的二级指标，请填写在此处**（行数可自行增加）：  指标描述：  增加理由： | | | | | | | | |

**表12：一级指标补充**

**如您认为有其他应当纳入的一级指标，请填写在下方（行数可自行增加），并说明增加理由。**

| **指标名称** | **指标描述** | **增加理由** |
| --- | --- | --- |
|  |  |  |
|  |  |  |
|  |  |  |

**最后，烦请专家填写下一页关键信息，便于我们联系您。**

**表13：专家信息表**

编号：_ _ _

1.您的年龄：____（周岁）

2.性别：____

3.最高学位：【单选】____

A.博士 B.硕士C.学士D.其他：____ _（请注明）

4.专业：【多选】____

A.预防医学或公共卫生B.临床医学C.国际卫生或全球卫生D.生物E.其他：______（请注明）

5.专业职称：【单选】____

A.正高级B.副高级C.中级D.初级E.其他：______（请注明）

6.当前职业：（可多选）____

A.生物学专家B.流行病学专家C.实验室人员 D.项目协调员或负责人E.政府官员（请注明领域：____）F.外科医生或专科医生 G.大学讲师或教授H.独立咨询顾问I.其他：______（请注明）

7.您目前的工作单位：【单选】____

A.公共卫生部门或疾病控制部门（技术机构）B.医院C.大专院校 D.政府部门E.国际组织F.企业/制药公司G.非政府组织H.基金会/捐助机构I.其他：______（请注明）

8.您通过何种方式参与全球卫生工作？【多选】____

A.国际会议B.国际培训C.合作研究或全球卫生项目D.其他：______（请注明）

9.您在全球卫生领域具有多少年的工作经验？【单选】____

A.在1年内B. 1-5年 C.在6-9年 D.10-20年 E.20年以上

10.您是否曾在中国以外的国家或地区工作过？包括短期进修、现场考察等【单选】____

A.是 B.否

**如回答是，请回答下列问题：**

11.您在这些国家或地区的工作时间有多久？（累计时长）【单选】____

A. 1年内B. 1-5年C. 6-9年D.10-20年E.超过20年

12.您所工作过的地区包括哪些？ 【多选】____

A.东亚和太平洋地区 B.欧洲 C.中亚地区D.南亚地区 E.中东和北非地区 F.撒哈拉以南非洲地区 G.拉丁美洲和加勒比地区

13.您的工作领域包括哪些？ 【多选】____

A.传染病（不包括HIV/AIDS）

B.HIV/AIDS

C.健康促进

D.伦理道德

E.妇幼健康

F.心理健康（不包括药物滥用）

G.慢性非传染性疾病（不包括心理健康）

H.生殖健康

I.卫生安全（生物安全）

J.药物滥用

K.外科手术和麻醉

L.全球卫生发展援助

M.其他：___________（请注明）

**填写结束，谢谢支持！**

**附件**

**CUGH全球卫生人员能力评价体系（供对照参考）**

| **Domains and Competencies** |
| --- |
|  |
| **DOMAIN: 1. Global Burden of Disease.**  Encompasses basic understandings of major causes of morbidity and mortality and theirvariations between high-, middle- and low-income regions, and with major public healthefforts to reduce health disparities globally. |
| 1a. Describe the major causes of morbidity and mortality around the world, and how therisk for disease varies with regions. |
| 1b. Describe major public health efforts to reduce disparities in global health (such asMillennium Development Goals and Global Fund to Fight AIDS, TB, and Malaria). |
| 1c. Validate the health status of populations using available data (e.g., public healthsurveillance data, vital statistics, registries, surveys, electronic health records, and healthplan claims data). |
| **DOMAIN: 2. Globalization of Health and Health Care**.  Focuses on understanding how globalization affects health, health systems, and thedelivery of health care. |
| 2a. Describe different national models or health systems for provision of health care andtheir respective effects on health and health care expenditure. |
| 2b. Describe how global trends in health care practice, commerce and culture, multinationalagreements, and multinational organizations contribute to the quality andavailability of health and health care locally and internationally. |
| 2c. Describe how travel and trade contribute to the spread of communicable andchronic diseases. |
| 2d. Describe general trends and influences in the global availability and movement ofhealth care workers. |
| **DOMAIN: 3. Social and Environmental Determinants of Health.**  Focuses on an understanding that social, economic, and environmental factors areimportant determinants of health and that health is more than the absence of disease. |
| 3a. Describe how cultural context influences perceptions of health and disease. |
| 3b. List major social and economic determinants of health and their effects on theaccess to and quality of health services and on differences in morbidity and mortality between and within countries. |
| 3c. Describe the relationship between access to and quality of water, sanitation, food,and air on individual and population health. |
| **DOMAIN: 4. Capacity Strengthening.**  “Capacity strengthening is sharing knowledge, skills, and resources for enhancing globalpublic health programs, infrastructure, and workforce to address current and future globalpublic health needs.” |
| 4a. Collaborate with a host or partner organization to assess the organization’s operationalcapacity. |
| 4b. Cocreate strategies with the community to strengthen community capabilities, andcontribute to reduction in health disparities and improvement of community health. |
| 4c. Integrate community assets and resources to improve the health of individualsandpopulations. |
| 4d. Identify methods for assuring program sustainability. |
| **DOMAIN: 5. Collaboration, Partnering, and Communication**.  “Collaborating and partnering is the ability to select, recruit, and work with a diverse rangeof global health stakeholders to advance research, policy, and practice goals, and to fosteropen dialogue and effective communication” with partners and within a team. |
| 5a. Include representatives of diverse constituencies in community partnerships and  foster interactive learning with these partners. |
| 5b. Demonstrate diplomacy and build trust with community partners. |
| 5c. Communicate joint lessons learned to community partners and globalconstituencies. |
| 5d. Exhibit interprofessional values and communication skills that demonstrate respectfor, and awareness of, the unique cultures, values, roles/responsibilities and expertiserepresented by other professionals and groups that work in global health. |
| 5e. Acknowledge one’s limitations in skills, knowledge, and abilities. |
| 5f. Apply leadership practices that support collaborative practice and teameffectiveness. |
| **DOMAIN: 6. Ethics**.  Encompasses the application of basic principles of ethics to global health issues and settings. |
| 6a. Demonstrate an understanding of and an ability to resolve common ethical issuesand challenges that arise when working within diverse economic, political, and culturalcontexts as well as when working with vulnerable populations and in low-resourcesettings to address global health issues. |
| 6b. Demonstrate an awareness of local and national codes of ethics relevant to one’sworking environment. |
| 6c. Apply the fundamental principles of international standards for the protection ofhuman subjects in diverse cultural settings. |
| **DOMAIN: 7: Professional Practice**.  Refers to activities related to the specific profession or discipline of the global healthpractitioner. (Domain definition proposed by members of the CUGH Global Health CompetencySubcommittee.) |
| 7a. Demonstrate integrity, regard, and respect for others in all aspects of professionalpractice. |
| 7b. Articulate barriers to health and health care in low-resource settings locally andinternationally. |
| 7c. Demonstrate the ability to adapt clinical or discipline-specific skills and practice in aresource-constrained setting. |
| **DOMAIN: 8. Health Equity and Social Justice.**  “Health equity and social justice is theframework for analyzing strategies to address health disparities across socially, demographically,or geographically defined populations.” |
| 8a. Apply social justice and human rights principles in addressing global healthproblems. |
| 8b. Implement strategies to engage marginalized and vulnerable populations in makingdecisions that affect their health and well-being. |
| 8c. Demonstrate a basic understanding of the relationships between health, humanrights, and global inequities. |
| 8d. Describe role of WHO in linking health and human rights, the Universal Declarationof Human Rights, International Ethical Guidelines for Biomedical Research InvolvingHuman Subjects. |
| 8e. Demonstrate a commitment to social responsibility. |
| 8f. Develop understanding and awareness of the health care workforce crisis in thedeveloping world, the factors that contribute to this, and strategies to address thisproblem. |
| **DOMAIN: 9. Program Management.**  “Program management is ability to design, implement, and evaluate global health programsto maximize contributions to effective policy, enhanced practice, and improved andsustainable health outcomes.” |
| 9a. Plan, implement, and evaluate an evidence-based program. |
| 9b. Apply project management techniques throughout program planning, implementation,and evaluation. |
| DOMAIN: 10. Sociocultural and Political Awareness.  “Sociocultural and political awareness is the conceptual basis with which to work effectivelywithin diverse cultural settings and across local, regional, national, and internationalpolitical landscapes.” |
| 10a. Describe the roles and relationships of the major entities influencing global healthand development. |
| **DOMAIN: 11. Strategic Analysis**.  “Strategic analysis is the ability to use systems thinking to analyze a diverse range ofcomplex and interrelated factors shaping health trends to formulate programs at the local,national, and international levels.” |
| 11a. Identify how demographic and other major factors can influence patterns ofmorbidity, mortality, and disability in a defined population. |
| 11b. Conduct a community health needs assessment. |
| 11c. Conduct a situation analysis across a range of cultural, economic, and healthcontexts. |
| 11d. Design context-specific health interventions based on situation analysis. |
