# Supplementary material for: Adaptation of the CUGH global health competency framework in the Chinese context: a mixed-methods study
Source: Glob Health Res Policy. 2023 Nov 2;8:46. doi: 10.1186/s41256-023-00327-w (PMC10621075; doi:10.1186/s41256-023-00327-w)
Supplement: Supplementary file 2 — Additional file 2: The questionnaire for the priority survey (in Chinese). [file 41256_2023_327_MOESM2_ESM.pdf]

# 中国公卫人员全球卫生胜任力优先指标调查问卷

各位专家，

在您的耐心帮助下，我们完成了“中国公卫人员全球卫生胜任力”指标的构建，对此我们表示由衷感谢！

为帮助教培人员今后更有针对性地开展能力提升活动，现邀请您：

1.根据您的工作经验，从已构建的10个胜任力指标中，选出您认为应当对中国公卫人员开展全球卫生培训的**优先指标**，并给出理由。

2.请您就提升中国公卫人员的全球卫生能力提出**培训建议**。

再次感谢您的支持！

中国疾控中心寄生虫病所全球卫生中心

2019年6月29日

---

**1. 请从已构建的10个胜任力指标中，选出您认为应当对中国公卫人员开展全球卫生培训的优先指标（数量不限）**【多选题】

- ☐ 指标1. 全球疾病负担
  了解高、中、低收入国家和地区的主要疾病负担的分布及原因。
- ☐ 指标2. 影响健康的主要因素
  了解社会、经济、环境和行为是健康的重要影响因素及其之间的相互作用，健康不仅是没有疾病，健康在所有相关政策中有所体现。
- ☐ 指标3. 全球化对人群健康、卫生系统和医疗服务的影响
  了解全球化如何影响人群健康、卫生系统和医疗服务。
- ☐ 指标4. 全球卫生领域的重要倡议和行动
  了解全球卫生的历史和重要倡议，能够辩证地思考全球卫生优先领域的演变以及当前的全球卫生行动。
- ☐ 指标5. 伦理、卫生公平和社会正义
  具备在运用基本伦理准则处理全球卫生问题的能力；具备运用卫生公平和社会正义分析框架处理不同社会环境、人口学或地理学特征人群所面对的健康不公平问题的能力。
- ☐ 指标6. 社会文化、政治意识和政策推动
  社会文化和政治意识是在不同文化背景下，在地方、区域、国家和国际政治环境中有效工作的重要前提。
- ☐ 指标7. 与全球卫生相关的个人基本素养和专业实践积累
  具备自身专业或学科有关活动所需要的必备素养、知识、技能和实践经验。
- ☐ 指标8. 能力加强
  能力加强 是指通过分享知识、技能和资源、完善全球公共卫生项目和基础设施、促进人力资源培养，来解决目前和未来的全球公共卫生需求。
- ☐ 指标9. 合作与沟通
  合作伙伴关系是为了改善人群健康，与各类全球卫生利益相关者开展合作，从而推动研究、影响卫生实践和政策制定的能力，以及与合作伙伴和团队内部建立开放式对话和有效沟通的能力。
- ☐ 指标10. 全球卫生项目管理
  项目管理能力包括设计、实施、督导和评估全球卫生项目，以最大程度促进全球卫生政策的可及性、有效性、可持续地改善卫生服务、促进健康。

**2. 您选择以上指标作为优先培训指标的理由：**

**3. 为更好地开展针对中国公卫人员从全球卫生能力培训，您的培训建议是：**

提示：可从教学设计、培训形式、内容、方法、师资等角度提供建议。

以下问题涉及到您的个人信息，该信息仅用于分析，我们将对您的信息进行保密。

**4. 您的性别：**

- ○ 男
- ○ 女

**5. 您的年龄段：**

- ○ 26~30
- ○ 31~40
- ○ 41~50
- ○ 51~60
- ○ 60以上

**6. 您的专业领域：**

**7. 您的专业职称：**

- ○ 正高级
- ○ 副高级
- ○ 中级
- ○ 其他 *

**8. 您目前的工作单位：**

- ○ 公共卫生部门或疾病控制部门
- ○ 医院
- ○ 高校
- ○ 政府部门
- ○ 国际组织
- ○ 企业
- ○ 非政府组织
- ○ 基金会/捐助机构
- ○ 其他 *

**9. 您在全球卫生领域具有多少年的工作经验？**

- ○ 1-5年
- ○ 6-9年
- ○ 10-20年
- ○ 20年以上

提交

举报

问卷星 提供技术支持
