# Supplementary material for: Adaptation of the CUGH global health competency framework in the Chinese context: a mixed-methods study
Source: Glob Health Res Policy. 2023 Nov 2;8:46. doi: 10.1186/s41256-023-00327-w (PMC10621075; doi:10.1186/s41256-023-00327-w)
Supplement: Supplementary file 3 — Additional file 3: The questionnaire for the priority survey (in English). [file 41256_2023_327_MOESM3_ESM.pdf]

Priority Global Health Competencies of Chinese Public Hea Practitioners

此问卷发布者已经通过实名认证

Dear experts,

Responding to the increasing globalization of health issues, more and more Chinese public healt h professionals engage in overseas health projects. However, there are no competencies identifie d to guide these training programmes, programme evaluation, job function delineation and conti nuous professional development assessments. To fill this gap, we conducted a three-round Delph i consultation, by consulting over 40 Chinese experts who are active in global health, used the US A-based Consortium of Universities for Global Health (CUGH)'s global health competencies as th e first draft.

Now, the *Global Health Competencies of Chinese Public Health Practitioners* has been finalized (e nclosed for your reference). Since you are an expert in global health and are very familiar with Chi nese public health practitioners, we are pleased to invite you to join us in this survey to:

1. Identify the priorir competencies for training Chinese public health practitioners and the ration al of your selection;
2. Offer your recommendations on the education and training to Chinese Public Health Practition ers, to strengthen their capacities in global health.

Thank you very much for your contribution!

Global Health Center
National Institute of Parasitic Diseases
Chinese Center for Disease Control and Prevention

*** 1. Please select the priority competencies (no limit) for training Chinese public health practitio ners to work in global health:【多选题】**

Please note that the target population is Chinese publich health practitioners, which requires you to thin k about their common weakness and the competencies could be strengthened from your perspective.

1. Global Burden of Disease
Understand the distribution and causes of major disease burden in high-, middle- and low-inc ome countries, territories and areas.

2. Determinants of Health
Understand that social, economic, environmental and behavioral factors, along with their inter actions, are important determinants of health. Health is more than the absence of disease, whi ch should be considered in all policies.

3. The impact of globalization on population health, health systems and healthcare
Understand how globalization affects health, health systems and health care

4. Major Global health initiatives and efforts
Knowledge of global health history and major initiatives, and the ability to think critically abou t the changing priorities on global health issues and current global health efforts.

5. Ethics, Health Equity and Social Justice
Ability to address global health issues with the basic principles of ethics; ability to address hea lth disparities by health equity and social justice frameworks across socially, demographically, or geographically defined populations.

6. Sociocultural, Political Awareness and Policy Promotion

Sociocultural and political awareness is the conceptual basis with which to work effectively within diverse cultural settings and across local, regional, national and international political landscapes.

7. Personal Competencies and Professional Practice

The necessary competencies, knowledge, skills and practical experience needed for professional activities.

8. Capacity strengthening

Capacity strengthening is sharing knowledge, skills and resources for enhancing global public health programs, infrastructure and workforce to address current and future global public health needs.

9. Collaboration, Partnering and Communication

Collaborating and partnering is the ability to select, recruit and work with a diverse range of global health stakeholders to advance research, policy and practice goals, and to foster open dialogue and effective communication with partners and within a team.

10. Program Management

Program management is ability to design, implement, supervise and evaluate global health programs to maximize contributions to effective policy, enhanced practice, and improved and sustainable health outcomes.

*** 2. Your reasons for choosing the above competencies as training priorities:**

*** 3. To facilitate the future advanced education for Chinese Public Health Practitioners, do you have any recommendations?**

Tip: Suggestions can be provided from the perspectives of training design, training method, content, and faculty, etc.

This is an anonymous survey. The following questions requires your personal information, which will be analyzed in our study only and will not be disclosed to a third party.

*** 4. Your gender:**

Male Female

*** 5. Your age:**

31~40 41~50 51~60 Over 60

*** 6. Your professional field:**

*** 7. Your position:**

Senior

Sub-senior

Medium

Other ________________ *

*** 8. You are currently employed by:**

public health sector/disease control department

hospital

college/university

government department

international organization

enterprise/pharmaceutical company

non-government organization

foundation/donor agency

other (please specify): ________________ *

*** 9. How long have you been working on(or work relating to) global health/international health?**

1-5 years

6-9 years

10-19 years

over 20 years

*** 10. How long have you been working with Chinese public health practitioners?**

1-5 years

6-9 years

10-19 years

over 20 years

提交

问卷星 提供技术支持
