# Supplementary material for: Adaptation of the CUGH global health competency framework in the Chinese context: a mixed-methods study
Source: Glob Health Res Policy. 2023 Nov 2;8:46. doi: 10.1186/s41256-023-00327-w (PMC10621075; doi:10.1186/s41256-023-00327-w)
Supplement: Supplementary file 4 — Additional file 4: Values of the Delphi experts’ judgment criterion (Ca) and familiarity (Cs) to each competency. [file 41256_2023_327_MOESM4_ESM.docx]

**Additional file 4. Values of the Delphi experts’ judgment criterion (C_a_) and familiarity (C_s_) to each competency**

Table 1 Values of the Delphi experts’ judgment criterion (C_a_)

| Judgment criterion (C_a_) | Degree of influence to experts' judgment | | |
| --- | --- | --- | --- |
|  | High | Medium | Low |
| Theory | 0.3 | 0.2 | 0.1 |
| Practice | 0.5 | 0.4 | 0.3 |
| Peers’ information | 0.1 | 0.1 | 0.1 |
| Intuition | 0.1 | 0.1 | 0.1 |
| Total | 1.0 | 0.8 | 0.6 |

Table 2 Values of the Delphi experts’ familiarity (C_s_)

| Familiarity (C_s_)* | Highest degree of familiar | High degree of familiar | Familiar | Low degree of familiar | Lower degree of familiar | Unfamiliar |
| --- | --- | --- | --- | --- | --- | --- |
|  | 1.0 | 0.8 | 0.6 | 0.4 | 0.2 | 0.0 |

*If the degree of familiarity lies in between two degrees, then the score could be 0.9, 0.7, 0.5, 0.3, and 0.1.
