# Supplementary material for: Adaptation of the CUGH global health competency framework in the Chinese context: a mixed-methods study
Source: Glob Health Res Policy. 2023 Nov 2;8:46. doi: 10.1186/s41256-023-00327-w (PMC10621075; doi:10.1186/s41256-023-00327-w)
Supplement: Supplementary file 5 — Additional file 5: The characteristics of the Delphi experts and the response rates. [file 41256_2023_327_MOESM5_ESM.docx]

**Additional file 5. The characteristics of the Delphi experts and the response rates**

Table 1 Characteristics of Delphi experts

| Characteristics |  | Number (N=48) | Percentage  (%) |
| --- | --- | --- | --- |
| Gender | Male | 31 | 64.58 |
|  | Female | 17 | 35.42 |
| Age | 30-39 | 13 | 27.08 |
|  | 40-49 | 11 | 22.92 |
|  | 50-59 | 17 | 35.42 |
|  | ≥60 | 7 | 14.58 |
| Professional title | Intermediate | 9 | 18.75 |
|  | Sub-senior | 11 | 22.92 |
|  | Senior | 28 | 58.33 |
| Education | Bachelor | 3 | 6.25 |
|  | Master | 14 | 29.17 |
|  | PhD | 31 | 64.58 |
| Years of work in global health | 2-5 | 12 | 25.00 |
|  | 6-9 | 15 | 31.25 |
|  | 10-20 | 13 | 27.08 |
|  | >20 | 8 | 16.67 |
| Employers | Public health sector | 13 | 27.08 |
|  | University | 21 | 43.75 |
|  | Government department (incl. research institutes and think tanks affiliated to government) | 7 | 14.58 |
|  | International organization | 2 | 4.17 |
|  | Non-government organization | 2 | 4.17 |
|  | Enterprise | 3 | 6.25 |
| Fields of research | Public health | 35 | 72.92 |
|  | International or global health | 20 | 41.67 |
|  | Health management, Health policy, Public policy | 7 | 14.58 |
|  | Clinical medicine | 4 | 8.33 |
|  | Biology | 1 | 2.08 |
|  | Pharmacy | 1 | 2.08 |
|  | Development studies | 2 | 4.17 |
|  | International Politics, Diplomacy | 2 | 4.17 |

Table 2 Experts’ response rate of three rounds of Delphi

|  | | 1^st^ round |  | 2^nd^ round | 3^rd^ round |
| --- | --- | --- | --- | --- | --- |
| Questionnaire delivered | | 53 |  | 47 | 42* |
| Questionnaire received | | 47 |  | 41 | 37 |
| Valid questionnaire | | 46 |  | 40 | 37 |
| Rate of response (%) | | 88.68% |  | 87.23% | 88.10% |
| Rate of valid questionnaire (%) | | 86.79% |  | 85.11% | 88.10% |
|  | * Note: One expert returned questionnaire only at the 3^rd^ round, while offered comments without returning the questionnaires during the 1^st^ and 2^nd^ round. | | | | |
