# Supplementary material for: Adaptation of the CUGH global health competency framework in the Chinese context: a mixed-methods study
Source: Glob Health Res Policy. 2023 Nov 2;8:46. doi: 10.1186/s41256-023-00327-w (PMC10621075; doi:10.1186/s41256-023-00327-w)
Supplement: Supplementary file 6 — Additional file 6: Experts’ scores and revisions of 1st round Delphi consultation. [file 41256_2023_327_MOESM6_ESM.docx]

## Additional file 6. Experts’ scores and revisions of 1^st^ round Delphi consultation

## Table 1 Experts’ scores of 1^st^ round Delphi consultation

| Item | Significance | | | |  | Feasibility | | | |
| --- | --- | --- | --- | --- | --- | --- | --- | --- | --- |
|  | Median | Mean | CV | Consensus (%score of≥3) |  | Median | Mean | CV | Consensus (%score of≥3) |
| DOMAIN: 1. Global Burden of Disease. Encompasses basic understandings of major causes of morbidity and mortality and their variations between high-, middle- and low-income regions, and with major public health efforts to reduce health disparities globally. | 5 | 4.63 | 0.14 | 100.00 |  | 5 | 4.41 | 0.16 | 100.00 |
| 1.1 Describe the major causes of morbidity and mortality around the world, and how the risk for disease varies with regions. | 5 | 4.58 | 0.14 | 100.00 |  | 4 | 4.26 | 0.20 | 97.83 |
| 1.2 Describe major public health efforts to reduce disparities in global health (such as Millennium Development Goals and Global Fund to Fight AIDS, TB, and Malaria). | 5 | 4.54 | 0.14 | 100.00 |  | 5 | 4.50 | 0.15 | 97.83 |
| 1.3 Validate the health status of populations using available data (e.g., public health surveillance data, vital statistics, registries, surveys, electronic health records, and health plan claims data). | 4.5 | 4.39 | 0.16 | 100.00 |  | 4 | 3.78 | 0.23 | 95.65 |
| DOMAIN: 2. Globalization of Health and Health Care. Focuses on understanding how globalization affects health, health systems, and the delivery of health care. | 5 | 4.63 | 0.11 | 100.00 |  | 4 | 3.83 | 0.21 | 97.83 |
| 2.1 Describe different national models or health systems for provision of health care and their respective effects on health and health care expenditure. | 5 | 4.42 | 0.17 | 97.78 |  | 4 | 3.80 | 0.24 | 91.11 |
| 2.2 Describe how global trends in health care practice, commerce and culture, multinational agreements, and multinational organizations contribute to the quality and availability of health and health care locally and internationally. | 4 | 4.22 | 0.19 | 95.65 |  | 3 | 3.39 | 0.29 | 82.61 |
| 2.3 Describe how travel and trade contribute to the spread of communicable and chronic diseases. | 4 | 4.10 | 0.21 | 93.48 |  | 4 | 3.54 | 0.29 | 84.78 |
| 2.4 Describe general trends and influences in the global availability and movement of health care workers. | 4 | 4.04 | 0.21 | 95.65 |  | 4 | 3.59 | 0.25 | 93.48 |
| DOMAIN: 3. Social and Environmental Determinants of Health. Focuses on an understanding that social, economic, and environmental factors are important determinants of health, and that health is more than the absence of disease. | 5 | 4.67 | 0.11 | 100.00 |  | 4 | 4.22 | 0.19 | 97.83 |
| 3.1 Describe how cultural context influences perceptions of health and disease. | 5 | 4.39 | 0.17 | 100.00 |  | 4 | 3.78 | 0.23 | 95.65 |
| 3.2 List major social and economic determinants of health and their effects on the access to and quality of health services and on differences in morbidity and mortality between and within countries. | 5 | 4.46 | 0.18 | 95.65 |  | 4 | 3.80 | 0.27 | 89.13 |
| 3.3 Describe the relationship between access to and quality of water, sanitation, food, and air on individual and population health. | 5 | 4.29 | 0.19 | 100.00 |  | 4 | 4.00 | 0.24 | 95.65 |
| DOMAIN: 4. Capacity Strengthening. “Capacity strengthening is sharing knowledge, skills, and resources for enhancing global public health programmes, infrastructure, and workforce to address current and future global public health needs.” | 5 | 4.64 | 0.13 | 100.00 |  | 4 | 3.99 | 0.23 | 93.33 |
| 4.1 Collaborate with a host or partner organization to assess the organization’s operational capacity. | 4 | 4.24 | 0.20 | 95.56 |  | 4 | 3.73 | 0.22 | 93.33 |
| 4.2 Cocreate strategies with the community to strengthen community capabilities, and contribute to reduction in health disparities and improvement of community health. | 5 | 4.44 | 0.17 | 100.00 |  | 4 | 3.79 | 0.23 | 91.11 |
| 4.3 Integrate community assets and resources to improve the health of individuals and populations. | 4 | 4.33 | 0.18 | 97.78 |  | 3 | 3.38 | 0.28 | 84.44 |
| 4.4 Identify methods for assuring programme sustainability. | 5 | 4.31 | 0.21 | 95.56 |  | 3 | 3.31 | 0.32 | 84.44 |
| DOMAIN: 5. Collaboration, Partnering, and Communication. “Collaborating and partnering is the ability to select, recruit, and work with a diverse range of global health stakeholders to advance research, policy, and practice goals, and to foster open dialogue and effective communication” with partners and within a team. | 5 | 4.87 | 0.07 | 100.00 |  | 4 | 4.27 | 0.20 | 97.78 |
| 5.1 Include representatives of diverse constituencies in community partnerships and foster interactive learning with these partners. | 5 | 4.57 | 0.14 | 100.00 |  | 4 | 3.78 | 0.26 | 89.13 |
| 5.2 Demonstrate diplomacy and build trust with community partners. | 5 | 4.54 | 0.16 | 100.00 |  | 4 | 3.74 | 0.27 | 86.96 |
| 5.3 Communicate joint lessons learned to community partners and global constituencies. | 5 | 4.71 | 0.11 | 100.00 |  | 4 | 4.09 | 0.22 | 95.65 |
| 5.4 Exhibit inter professional values and communication skills that demonstrate respect for, and awareness of, the unique cultures, values, roles/responsibilities and expertise represented by other professionals and groups that work in global health. | 5 | 4.72 | 0.12 | 100.00 |  | 4 | 3.93 | 0.26 | 86.96 |
| 5.5 Acknowledge one’s limitations in skills, knowledge, and abilities. | 5 | 4.37 | 0.18 | 100.00 |  | 4 | 3.83 | 0.24 | 95.65 |
| 5.6 Apply leadership practices that support collaborative practice and team effectiveness. | 5 | 4.57 | 0.15 | 97.83 |  | 4 | 3.96 | 0.21 | 97.83 |
| DOMAIN: 6. Ethics. Encompasses the application of basic principles of ethics to global health issues and settings. | 5 | 4.63 | 0.11 | 100.00 |  | 4 | 3.80 | 0.26 | 89.13 |
| 6.1 Demonstrate an understanding of and an ability to resolve common ethical issues and challenges that arise when working within diverse economic, political, and cultural contexts as well as when working with vulnerable populations and in low-resource settings to address global health issues. | 5 | 4.65 | 0.11 | 100.00 |  | 4 | 3.65 | 0.23 | 91.30 |
| 6.2 Demonstrate an awareness of local and national codes of ethics relevant to one’s working environment. | 5 | 4.74 | 0.09 | 100.00 |  | 4 | 3.87 | 0.25 | 91.30 |
| 6.3 Apply the fundamental principles of international standards for the protection of human subjects in diverse cultural settings. | 5 | 4.61 | 0.12 | 100.00 |  | 4 | 3.59 | 0.30 | 78.26 |
| DOMAIN: 7: Professional Practice. Refers to activities related to the specific profession or discipline of the global health professional. (Domain definition proposed by members of the CUGH Global Health Competency Subcommittee.) | 5 | 4.78 | 0.11 | 100.00 |  | 5 | 4.37 | 0.17 | 100.00 |
| 7.1 Demonstrate integrity, regard, and respect for others in all aspects of professional practice. | 5 | 4.78 | 0.10 | 100.00 |  | 4 | 4.17 | 0.22 | 93.48 |
| 7.2 Articulate barriers to health and health care in low-resource settings locally and internationally. | 5 | 4.57 | 0.15 | 100.00 |  | 4 | 3.91 | 0.24 | 93.48 |
| 7.3 Demonstrate the ability to adapt clinical or discipline-specific skills and practice in are source-constrained setting. | 5 | 4.52 | 0.16 | 100.00 |  | 3.5 | 3.67 | 0.28 | 89.13 |
| DOMAIN: 8. Health Equity and Social Justice. “Health equity and social justice is the framework for analyzing strategies to address health disparities across socially, demographically, or geographically defined populations.” | 5 | 4.52 | 0.14 | 100.00 |  | 4 | 3.85 | 0.20 | 97.83 |
| 8.1 Apply social justice and human rights principles in addressing global health problems. | 5 | 4.46 | 0.17 | 97.83 |  | 4 | 3.65 | 0.25 | 91.30 |
| 8.2 Implement strategies to engage marginalized and vulnerable populations in making decisions that affect their health and well-being. | 4.5 | 4.39 | 0.16 | 97.83 |  | 4 | 3.59 | 0.24 | 89.13 |
| 8.3 Demonstrate a basic understanding of the relationships between health, human rights, and global inequities. | 5 | 4.41 | 0.17 | 100.00 |  | 4 | 3.76 | 0.27 | 89.13 |
| 8.4 Describe role of WHO in linking health and human rights, the Universal Declaration of Human Rights, International Ethical Guidelines for Biomedical Research Involving Human Subjects. | 4 | 4.02 | 0.23 | 93.48 |  | 4 | 3.74 | 0.28 | 84.78 |
| 8.5 Demonstrate a commitment to social responsibility. | 5 | 4.65 | 0.12 | 100.00 |  | 4 | 3.96 | 0.24 | 93.48 |
| 8.6 Develop understanding and awareness of the health care workforce crisis in the developing world, the factors that contribute to this, and strategies to address this problem. | 4 | 4.17 | 0.21 | 95.65 |  | 3 | 3.46 | 0.27 | 84.78 |
| DOMAIN: 9. Programme Management. “Programme management is ability to design, implement, and evaluate global health programmes to maximize contributions to effective policy, enhanced practice, and improved and sustainable health outcomes.” | 5 | 4.76 | 0.11 | 100.00 |  | 5 | 4.35 | 0.19 | 97.83 |
| 9.1 Plan, implement, and evaluate an evidence-based programme. | 5 | 4.72 | 0.12 | 100.00 |  | 4.5 | 4.33 | 0.19 | 95.65 |
| 9.2 Apply project management techniques throughout programme planning, implementation, and evaluation. | 5 | 4.74 | 0.14 | 97.83 |  | 5 | 4.39 | 0.18 | 97.83 |
| DOMAIN: 10. Sociocultural and Political Awareness. “Sociocultural and political awareness is the conceptual basis with which to work effectively within diverse cultural settings and across local, regional, national, and international political landscapes.” | 5 | 4.47 | 0.14 | 100.00 |  | 4 | 3.65 | 0.21 | 93.48 |
| 10.1 Describe the roles and relationships of the major entities influencing global health and development. | 4 | 4.27 | 0.17 | 100.00 |  | 4 | 3.63 | 0.23 | 93.48 |
| DOMAIN: 11. Strategic Analysis. “Strategic analysis is the ability to use systems thinking to analyze a diverse range of complex and interrelated factors shaping health trends to formulate programmes at the local, national, and international levels.” | 5 | 4.57 | 0.14 | 100.00 |  | 4 | 3.60 | 0.23 | 91.30 |
| 11.1 Identify how demographic and other major factors can influence patterns of morbidity, mortality, and disability in a defined population. | 5 | 4.41 | 0.17 | 97.83 |  | 4 | 3.70 | 0.24 | 93.48 |
| 11.2 Conduct a community health needs assessment. | 5 | 4.39 | 0.16 | 100.00 |  | 4 | 4.02 | 0.25 | 93.48 |
| 11.3 Conduct a situation analysis across a range of cultural, economic, and health contexts. | 5 | 4.48 | 0.16 | 100.00 |  | 4 | 3.74 | 0.21 | 95.65 |
| 11.4 Design context-specific health interventions based on situation analysis. | 5 | 4.50 | 0.17 | 97.83 |  | 4 | 3.83 | 0.25 | 93.48 |

Revisions of the 1^st^ round Delphi consultation

1. 11 new secondary competencies were added to Domain 1,3,5,8, and10:

- Two new secondary competencies were added under ‘1. Global Burden of Disease’— ‘Describe the key disease burden indicators’ and ‘Describe the major health issues of vulnerable populations and the key issues in the global health arena’.
- Three new secondary competencies were added under ‘3. Social and Environmental Determinants of Health’— ‘Describe the impact of R&D, production and access on global health from a global public health product perspective and be aware of the intellectual property rights of health technologies’, ‘Describe the behavioral factors of health determinants’, and ‘Describe major intervention strategies of public health issues’.
- One new secondary competency was added under ‘5. Collaboration, Partnering, and Communication’— ‘Communicate effectively in foreign language (i.e. English, French, Spanish, Arabic, or any other local language) \ and the ability to work cross-culturally.’
- One new secondary competency was added under ‘8. Health Equity and Social Justice’— ‘Understand the barriers to access and equity of primary health care services for populations in developing countries.’
- Four new secondary competencies were added under ‘10. Sociocultural and Political Awareness’— ‘Describe China's basic national conditions, roles and policies in global health under new situations.’, ‘Awareness of the information of politics, culture, environment, society, religion, law, diplomacy and national security.’, ‘Capabilities of participation in national health security’, and ‘The ability to shape policy’.

1. competency 1.2 ‘Describe major public health efforts to reduce disparities in global health’ was listed as a new domain and two new secondary competencies were added: ‘Describe the major global health efforts’ and ‘Describe the global health history and its development’.
2. competency 4.4 ‘Identify methods for assuring programme sustainability’ was moved to Domain 9 ‘Programme Management’.
3. five new competencies were left unclassified as a new domain: ‘Fast learning ability’, ‘Emotion management skills’, ‘Ability to spread knowledge and skills’, ‘The ability to organize team members to accumulate professional knowledge’, ‘Demonstrate integrity, regard, and respect for others in all aspects of professional practice (7.1)’.
