# Supplementary material for: Adaptation of the CUGH global health competency framework in the Chinese context: a mixed-methods study
Source: Glob Health Res Policy. 2023 Nov 2;8:46. doi: 10.1186/s41256-023-00327-w (PMC10621075; doi:10.1186/s41256-023-00327-w)
Supplement: Supplementary file 7 — Additional file 7: Experts’ scores and revisions of 2nd round Delphi consultation. [file 41256_2023_327_MOESM7_ESM.docx]

## Additional file 7. Experts’ scores and revisions of 2^nd^ round Delphi consultation

## Table 1 Experts’ scores of 2^nd^ round Delphi consultation

| Item | Significance | | | |  | Feasibility | | | |
| --- | --- | --- | --- | --- | --- | --- | --- | --- | --- |
|  | Median | Mean | CV | Consensus (%score of≥3) |  | Median | Mean | CV | Consensus (%score of≥3) |
| DOMAIN: 1. Global Burden of Disease. Encompasses basic understandings of major causes of morbidity and mortality and their variations between high-, middle- and low-income regions. | 5 | 4.64 | 0.15 | 97.50 |  | 5 | 4.43 | 0.16 | 100.00 |
| 1.1 Describe the major causes of morbidity and mortality around the world, and how the risk for disease varies with regions and population. | 5 | 4.42 | 0.18 | 97.44 |  | 4 | 4.18 | 0.22 | 94.87 |
| 1.2 Validate the health status of populations using available data worldwide. E.g., public health surveillance data, vital statistics, registries, surveys, electronic health records, World Health Statistics, national policies, scientific literatures, and health plan claims data. | 5 | 4.59 | 0.15 | 97.50 |  | 4 | 4.26 | 0.19 | 97.50 |
| 1.3 Describe the key disease burden indicators and global disease burden  E.g., global disease burden and its development trends, main classification characteristics, main influencing factors, major limitations of existing intervention strategies, innovative intervention strategy design, etc. | 5 | 4.43 | 0.16 | 97.50 |  | 4 | 4.05 | 0.19 | 97.50 |
| 1.4 Describe the major health issues of vulnerable populations and the key issues in global health arena. E.g., non-communicable diseases, emerging infectious diseases, and mental health. | 5 | 4.45 | 0.16 | 97.44 |  | 4 | 4.09 | 0.20 | 97.50 |
| DOMAIN 2. Global Public Health Initiatives and Efforts | 5 | 4.76 | 0.12 | 97.50 |  | 5 | 4.68 | 0.10 | 100.00 |
| 2.1 Describe the major global health initiatives (such as health targets in the 2030 Agenda for Sustainable Development). | 5 | 4.70 | 0.14 | 97.50 |  | 5 | 4.75 | 0.10 | 100.00 |
| 2.2 Describe the major global health efforts, include main activities, financing and stakeholders. | 5 | 4.56 | 0.14 | 97.50 |  | 4 | 4.08 | 0.17 | 100.00 |
| 2.3 Describe the global health history and its development, especially the work history of developing countries and its current situation, and the ability to analyze and learn from the past. | 4 | 4.13 | 0.19 | 97.50 |  | 4 | 4.09 | 0.19 | 97.50 |
| DOMAIN: 3. Globalization of Health and Health Care. Understand how globalization affects health, health systems, and the delivery of health care. | 5 | 4.64 | 0.15 | 97.50 |  | 4 | 4.08 | 0.16 | 100.00 |
| 3.1 Describe different national models or health systems for provision of health care and their respective effects on health and health care expenditure. | 4 | 4.33 | 0.16 | 100.00 |  | 4 | 3.78 | 0.24 | 95.00 |
| 3.2 Describe how global trends in health care practice, commerce and culture, multinational agreements, and multinational organizations contribute to the quality and availability of health and health care locally and internationally. | 4 | 4.24 | 0.14 | 100.00 |  | 4 | 3.70 | 0.22 | 97.50 |
| 3.3 Describe how globalization influence national health security and how travel and trade contribute to the spread of communicable and chronic diseases. | 5 | 4.39 | 0.17 | 97.50 |  | 4 | 3.94 | 0.22 | 97.50 |
| 3.4 Describe general trends and influences in the global availability and movement of health care workers; understand and aware the health care workforce crisis in the developing world, the factors that contribute to this, and strategies to address this problem. | 4 | 4.13 | 0.18 | 100.00 |  | 4 | 3.78 | 0.21 | 97.50 |
| DOMAIN: 4. Determinants of Health (Social, Environmental, and Behavioral) Understand that social, economic, and environmental factors are important determinants of health, and that health is more than the absence of disease. | 5 | 4.74 | 0.14 | 97.50 |  | 4 | 4.30 | 0.18 | 100.00 |
| 4.1 Describe how cultural context and education influences perceptions of health and disease. | 5 | 4.38 | 0.16 | 100.00 |  | 4 | 4.12 | 0.19 | 97.44 |
| 4.2 List major social determinants of health and their effects on the access to and quality of health services and on differences in morbidity and mortality between and within countries. | 4 | 4.29 | 0.16 | 100.00 |  | 4 | 3.85 | 0.23 | 94.87 |
| 4.3 List major economic determinants of health and their effects on the access to and quality of health services and on differences in morbidity and mortality between and within countries. | 4 | 4.29 | 0.17 | 97.44 |  | 4 | 3.95 | 0.21 | 97.44 |
| 4.4 Describe the impact of R&D, production and access on global health from a global public health product perspective, and be aware of the intellectual property rights of health technologies | 4 | 4.03 | 0.19 | 97.50 |  | 4 | 3.65 | 0.27 | 90.00 |
| 4.5 Describe the relationship between access to and quality of water, sanitation, food, and air on individual and population health. | 4 | 4.20 | 0.20 | 97.50 |  | 4 | 4.01 | 0.23 | 97.50 |
| 4.6 Describe the behavioral factors of health determinants. E.g., incidence of HIV/AIDS vs. drug use, male homosexual behavior, and multiple sexual partners, chronic disease vs. smoking and lack of exercise, etc. | 5 | 4.49 | 0.17 | 97.50 |  | 4 | 4.20 | 0.19 | 97.50 |
| 4.7 Describe major intervention strategies of public health issues, including the existing interventions, implemented interventions, planned interventions, to-be changed or improved interventions, to-be innovated interventions, etc. | 5 | 4.45 | 0.17 | 97.44 |  | 4 | 4.03 | 0.19 | 100.00 |
| DOMAIN: 5. Capacity Strengthening. Capacity strengthening is sharing knowledge, skills, and resources for enhancing global public health programmes, infrastructure, and workforce to address current and future global public health needs. | 5 | 4.68 | 0.11 | 100.00 |  | 4 | 4.00 | 0.21 | 97.44 |
| 5.1 Collaborate with a host or partner organization to assess the organization’s operational capacity. | 5 | 4.46 | 0.15 | 100.00 |  | 4 | 4.05 | 0.22 | 97.50 |
| 5.2 Cocreate strategies with the community to strengthen community capabilities, and contribute to reduction in health disparities and improvement of community health. | 5 | 4.64 | 0.11 | 100.00 |  | 4 | 3.85 | 0.22 | 90.00 |
| 5.3 In the case of being empowered, integrate community assets and resources to improve the health of individuals and populations. | 5 | 4.43 | 0.16 | 100.00 |  | 4 | 3.79 | 0.26 | 87.50 |
| DOMAIN: 6. Collaboration, Partnering, and Communication. Collaborating and partnering is the ability to select, recruit, and work with a diverse range of global health stakeholders to advance research, policy, and practice goals, and to foster open dialogue and effective communication with partners and within a team. | 5 | 4.86 | 0.08 | 100.00 |  | 4 | 4.30 | 0.16 | 100.00 |
| 6.1 Include representatives of diverse constituencies in community partnerships and foster interactive learning with these partners. Communicate joint lessons learned to community partners and global constituencies. | 5 | 4.76 | 0.10 | 100.00 |  | 4 | 4.18 | 0.16 | 100.00 |
| 6.2 Demonstrate diplomacy and build trust with community partners. Exhibit inter professional values and communication skills that demonstrate respect for, and awareness of, the unique cultures, values, roles/responsibilities and expertise represented by other professionals and groups that work in global health. | 5 | 4.68 | 0.11 | 100.00 |  | 4 | 3.81 | 0.23 | 90.00 |
| 6.3 Acknowledge one’s limitations in skills, knowledge, and abilities. Apply leadership practices that support collaborative practice and team effectiveness. | 5 | 4.48 | 0.16 | 100.00 |  | 4 | 4.05 | 0.19 | 97.50 |
| 6.4 Communicate effectively in foreign language (i.e. English, French, Spanish, Arabic, or any other local language) and the ability to work cross-culturally. | 5 | 4.76 | 0.12 | 97.50 |  | 5 | 4.23 | 0.23 | 92.31 |
| DOMAIN: 7. Ethics. Encompasses the application of basic principles of ethics to global health issues and settings. | 5 | 4.88 | 0.07 | 100.00 |  | 4 | 4.09 | 0.19 | 97.50 |
| 7.1 Demonstrate an understanding of and an ability to resolve common ethical issues and challenges that arise when working within diverse economic, political, and cultural and religious contexts as well as when working with vulnerable populations and in low-resource settings to address global health issues. | 5 | 4.74 | 0.10 | 100.00 |  | 4 | 3.95 | 0.21 | 97.50 |
| 7.2 Demonstrate an awareness of local and national codes of ethics relevant to one’s working environment. | 5 | 4.68 | 0.12 | 100.00 |  | 4 | 4.08 | 0.22 | 95.00 |
| 7.3 Apply the fundamental principles of international standards for the protection of human subjects in diverse cultural settings. E.g., World Medical Association Declaration of Helsinki, the relevant ethical standards of WHO. | 5 | 4.50 | 0.15 | 100.00 |  | 4 | 3.91 | 0.23 | 92.50 |
| DOMAIN: 8. Professional Practice. Refers to activities related to the specific profession or discipline of the global health professional. | 5 | 4.68 | 0.13 | 97.50 |  | 4 | 4.35 | 0.15 | 100.00 |
| 8.1 Articulate barriers to health and health care in low-resource settings locally and internationally. | 5 | 4.51 | 0.14 | 97.44 |  | 4 | 3.86 | 0.21 | 92.31 |
| 8.2 Demonstrate the ability to adapt discipline-specific skills and practice in are source-constrained setting. | 5 | 4.55 | 0.16 | 97.50 |  | 4 | 3.80 | 0.22 | 95.00 |
| DOMAIN: 9. Health Equity and Social Justice. Health equity and social justice is the framework for analyzing strategies to address health disparities across socially, demographically, or geographically defined populations. | 5 | 4.53 | 0.13 | 100.00 |  | 4 | 3.85 | 0.19 | 100.00 |
| 9.1 Apply social justice and human rights principles in addressing global health problems. | 5 | 4.54 | 0.15 | 97.50 |  | 4 | 3.83 | 0.22 | 95.00 |
| 9.2 Understand the barriers to access and equity of primary health care services for populations in developing countries. | 5 | 4.53 | 0.16 | 97.50 |  | 4 | 4.21 | 0.20 | 100.00 |
| 9.3 Implement strategies to engage marginalized and vulnerable populations in making decisions that affect their health and well-being. | 5 | 4.49 | 0.15 | 97.50 |  | 4 | 3.63 | 0.26 | 90.00 |
| 9.4 Demonstrate a basic understanding of the relationships between health, human rights, and global inequities. | 4 | 4.16 | 0.16 | 97.50 |  | 4 | 3.89 | 0.22 | 95.00 |
| 9.5 Describe role of WHO in linking health and human rights, the Universal Declaration of Human Rights, International Ethical Guidelines for Biomedical Research Involving Human Subjects. | 4 | 4.10 | 0.17 | 100.00 |  | 4 | 3.99 | 0.23 | 92.50 |
| 9.6 Demonstrate a commitment to social responsibility. | 5 | 4.70 | 0.13 | 97.50 |  | 4.25 | 4.29 | 0.18 | 100.00 |
| DOMAIN: 10. Programme Management. Programme management is ability to design, implement, and evaluate global health programmes to maximize contributions to effective policy, enhanced practice, and improved and sustainable health outcomes. | 5 | 4.78 | 0.09 | 100.00 |  | 4 | 4.31 | 0.16 | 97.50 |
| 10.1 Plan, implement, and evaluate an evidence-based programme. | 5 | 4.69 | 0.12 | 100.00 |  | 4 | 4.19 | 0.20 | 95.00 |
| 10.2 Apply project management techniques throughout programme planning, implementation, and evaluation. | 5 | 4.70 | 0.11 | 100.00 |  | 4 | 4.15 | 0.19 | 97.50 |
| 10.3 Identify methods for assuring programme sustainability. | 4.75 | 4.41 | 0.15 | 100.00 |  | 3.5 | 3.65 | 0.24 | 94.87 |
| DOMAIN: 11. Sociocultural and Political Awareness. Sociocultural and political awareness is the conceptual basis with which to work effectively within diverse cultural settings and across local, regional, national, and international political landscapes. | 5 | 4.47 | 0.15 | 97.44 |  | 4 | 3.82 | 0.18 | 100.00 |
| 11.1 Describe the roles and relationships of the major entities influencing global health and development; describe the various global health actors, the role of different types of actors in global health governance, their contribution and challenges, and coping strategies. | 4 | 4.21 | 0.17 | 100.00 |  | 4 | 3.66 | 0.18 | 97.50 |
| 11.2 Describe China's basic national conditions, history of global health activities, experience and lessons. China's role and its functions, and policies in global health under new situations. | 5 | 4.45 | 0.17 | 97.50 |  | 4 | 4.23 | 0.16 | 0.99 |
| 11.3 Awareness of the information of politics, culture, environment, society, religion, law, diplomacy and national security. | 5 | 4.36 | 0.19 | 97.50 |  | 4 | 3.83 | 0.20 | 97.50 |
| 11.4 Capabilities of participation in national health security, including global health and safety assessment, practice, emergency response and other capabilities. | 4.25 | 4.24 | 0.20 | 97.50 |  | 4 | 3.85 | 0.19 | 100.00 |
| 11.5 The ability to shape policy. Familiar with the policy procedures and political characteristics of the target country, with the ability to translate data, evidence and work plans into policy statements, policy documents and the implementation of relevant policies in a complex policy environment. | 4.5 | 4.38 | 0.16 | 100.00 |  | 4 | 3.58 | 0.23 | 92.50 |
| DOMAIN: 12. Strategic Analysis. Strategic analysis is the ability to use systems thinking to analyze a diverse range of complex and interrelated factors shaping health trends to formulate programmes at the local, national, and international levels. | 5 | 4.61 | 0.14 | 100.00 |  | 4 | 3.84 | 0.20 | 95.00 |
| 12.1 Identify how demographic and other major factors can influence patterns of morbidity, mortality, and disability in a defined population. | 5 | 4.53 | 0.17 | 97.44 |  | 4 | 3.92 | 0.22 | 97.44 |
| 12.2 Conduct a situation analysis across a range of cultural, economic, and health contexts. | 5 | 4.60 | 0.14 | 100.00 |  | 4 | 3.94 | 0.22 | 97.50 |
| 12.3 Design context-specific health interventions based on situation analysis. | 5 | 4.56 | 0.14 | 100.00 |  | 4 | 3.98 | 0.21 | 95.00 |
| DOMAIN: 13. Unclassified competencies | - | - | - | - |  | - | - | - | - |
| 13.1 Fast learning ability. Get the most up-to-date and comprehensive information quickly; quickly locate the knowledge points you need for your work from the vast amount of information | 5 | 4.65 | 0.12 | 100.00 |  | 4 | 4.06 | 0.19 | 100.00 |
| 13.2 Emotion management (compression and conflict response) skills. No personality defects, strong psychological endurance and coping skills | 5 | 4.61 | 0.13 | 100.00 |  | 4 | 4.03 | 0.19 | 100.00 |
| 13.3 Ability to spread knowledge and skills. | 4.25 | 4.36 | 0.16 | 100.00 |  | 4 | 4.00 | 0.18 | 100.00 |
| 13.4 The ability to organize team members to accumulate professional knowledge. | 5 | 4.40 | 0.16 | 100.00 |  | 4 | 4.03 | 0.18 | 100.00 |
| 13.5 Demonstrate integrity, regard, and respect for others in all aspects of professional practice. | 5 | 4.60 | 0.14 | 100.00 |  | 4.75 | 4.35 | 0.18 | 97.50 |

Revisions of 2^nd^ round Delphi consultation

DOMAIN: 1. Global Burden of Disease.

- Combination: ‘1.1 Describe the major causes of morbidity and mortality around the world, and how the risk for disease varies with regions and population.’ and ‘1.3 Describe the key disease burden indicators and global disease burden’ be combined as ‘1.1 Ability to analyze the morbidity and mortality of major disease around the world, and the indicators and trends of disease burden.’
- During the 1^st^ round Delphi, one expert suggests adding ‘Can correctly use data to analyze, explain and address problems’; during the 2^nd^ round Delphi, three experts suggest that the competencies shall not be limited to ‘describe’ but the ‘ability to use the information to analyze problems’. Therefore, the competencies under this domain were adjusted— ‘describe’ were replaced by ‘obtain, analyze, and apply’.

DOMAIN 2. Global Public Health Initiatives and Efforts

- Move: ‘4.7 Describe major intervention strategies of public health issues’ was moved to DOMAIN 2 as ‘2.3 Describe major intervention strategies, best practices, limitation of current interventions, and innovation strategies of public health issues.’

DOMAIN: 3. Globalization of Health and Health Care

- Combination: ‘3.3 Describe how globalization influence national health security and how travel and trade contribute to the spread of communicable and chronic diseases.’ and ‘3.4 Describe general trends and influences in the global availability and movement of health care workers; understand and aware the health care workforce crisis in the developing world, the factors that contribute to this, and strategies to address this problem.’ were combined as a footnote of ‘3.2 Describe how global trends in health care practice, commerce and culture, multinational agreements, and multinational organizations contribute to the quality and availability of health and health care locally and internationally.’

DOMAIN: 4. Determinants of Health.

- Combination: according to experts’ suggestion and the literature review, the ‘economic determinants’ and the ‘social determinants’ were combined.
- Move: ‘4.4 Describe the impact of R&D, production and access on global health from a global public health product perspective and be aware of the intellectual property rights of health technologies’ moved to DOMAIN 3 as 3.3.
- Move: 4.7 moved to 2.3.

DOMAIN: 5. Capacity Strengthening

- Edits were made to increase comprehensiveness.

DOMAIN: 6. Collaboration, Partnering, and Communication

- Combination: ‘6.3 Acknowledge one’s limitations in skills, knowledge, and abilities. Apply leadership practices that support collaborative practice and team effectiveness.’ combine with ‘13.1 Fast learning ability’.
- Separate & Combination: Separate ‘Apply leadership practices that support collaborative practice and team effectiveness.’ from 6.3. Moved ‘13.4 The ability to organize team members to accumulate professional knowledge.’ to the footnote of this competency.
- Move: Moved ‘13.3 Ability to spread knowledge and skills.’ to DOMAIN 6 as 6.4.
- Move: Moved and rephrased ‘6.4 Communicate effectively in foreign language…’ under DOMAIN 13

DOMAIN: 7. Ethics

- Combination: Combined with ‘DOMAIN: 9. Health Equity and Social Justice’ as they share similarities in essence. Combined ‘9.1 Apply social justice and human rights principles in addressing global health problems.’ and ‘7.1’; 9.2, 9.3, 9.4 listed as 7.4, 7.5, 7.6, 7.7; 9.5 combined with 7.3.

DOMAIN: 8. Professional Practice

- Move & Combination: Combined 8.1 to 12.1 to avoid redundancy.

DOMAIN: 9. Health Equity and Social Justice

- Combined with ‘DOMAIN: 7. Ethics’.

DOMAIN: 10. Programme Management

- Combination & Move: Combined 12.1 and 12.2, and moved under DOMAIN 10 as ‘Strategic analysis. According to the health determinants, conduct a need assessment and situation analysis across a range of cultural, economic, and health contexts.’
- Combination: Combine ‘12.3 Design context-specific health interventions based on situation analysis.’ with ‘10.1 Plan, implement, and evaluate an evidence-based programme.’

DOMAIN: 11. Sociocultural and Political Awareness

- Complement: Add ‘Policy Promotion’ to DOMAIN 11 to include ‘11.5 The ability to shape policy.’
- Move: Moved ‘11.4 Capabilities of participation in national health security, including global health and safety assessment, practice, emergency response and other capabilities.’ to 8.2.

DOMAIN: 12. Strategic Analysis

- Moved under DOMAIN: 10. ‘Programme Management’ as strategic analysis was recognized as a component of programme management.

DOMAIN: 13. Unclassified competencies

- Rename: Renamed DOMAIN 13 as ‘Personal Competencies’ and move relating competencies under this domain.
- Move: Moved and rephrased ‘6.4 Communicate effectively in foreign language…’ under DOMAIN 13 as ‘Communicate effectively in the official language of the target context…’.
- Combination: Combined ‘6.3 Acknowledge one’s limitations in skills, knowledge, and abilities…’ with ‘13.1 Fast learning ability…’.
- Move: Moved ‘13.3 Ability to spread knowledge and skills.’ and ‘13.4 The ability to organize team members to accumulate professional knowledge.’ to ‘DOMAIN: 6. Collaboration, Partnering, and Communication’.
