# Supplementary material for: Adaptation of the CUGH global health competency framework in the Chinese context: a mixed-methods study
Source: Glob Health Res Policy. 2023 Nov 2;8:46. doi: 10.1186/s41256-023-00327-w (PMC10621075; doi:10.1186/s41256-023-00327-w)
Supplement: Supplementary file 8 — Additional file 8: Experts’ scores of 3rd round Delphi consultation and results of the panel discussion. [file 41256_2023_327_MOESM8_ESM.docx]

## **Additional file 8.** Experts’ scores of 3^rd^ round Delphi consultation and results of the panel discussion

Table 1 Experts’ scores of 3^rd^ round Delphi consultation

| Item | Significance | | | |  | Feasibility | | | |
| --- | --- | --- | --- | --- | --- | --- | --- | --- | --- |
|  | Median | Mean | CV | Consensus (%score of≥3) |  | Median | Mean | CV | Consensus (%score of≥3) |
| DOMAIN: 1. Global Burden of Disease. Encompasses basic understandings of major causes of morbidity and mortality and their variations between high-, middle- and low-income regions. | 5 | 4.66 | 0.14 | 100.00 |  | 5 | 4.46 | 0.18 | 97.22 |
| 1.1 Ability to analyze the morbidity and mortality of major disease around the world, and the indicators and trends of disease burden. | 5 | 4.59 | 0.16 | 97.30 |  | 5 | 4.39 | 0.16 | 100.00 |
| 1.2 Validate the health status of populations using available data worldwide. | 5 | 4.57 | 0.17 | 97.30 |  | 5 | 4.24 | 0.20 | 100.00 |
| 1.3 Describe the major health issues of vulnerable populations and the key issues in global health arena. E.g., non-communicable diseases, emerging infectious diseases, and mental health. | 5 | 4.59 | 0.12 | 100.00 |  | 4 | 4.22 | 0.18 | 100.00 |
| DOMAIN 2. Global Public Health Initiatives and Efforts | 5 | 4.62 | 0.12 | 100.00 |  | 5 | 4.50 | 0.14 | 100.00 |
| 2.1 Describe the major global health initiatives (such as health targets in the 2030 Agenda for Sustainable Development). | 5 | 4.51 | 0.14 | 100.00 |  | 5 | 4.55 | 0.13 | 100.00 |
| 2.2 Describe the major global health efforts, include main activities, financing and stakeholders. | 5 | 4.43 | 0.15 | 100.00 |  | 5 | 4.51 | 0.13 | 100.00 |
| 2.3 Describe major intervention strategies, best practices, limitation of current interventions, and innovation strategies of public health issues. | 5 | 4.49 | 0.14 | 100.00 |  | 4.75 | 4.21 | 0.22 | 97.22 |
| 2.4 Describe the global health history and its development, especially the work history of developing countries and its current situation, and the ability to analyze and learn from the past. | 4 | 4.09 | 0.17 | 100.00 |  | 4 | 3.99 | 0.21 | 94.59 |
| DOMAIN: 3. Globalization of Health and Health Care. Understand how globalization affects health, health systems, and the delivery of health care. | 5 | 4.51 | 0.16 | 100.00 |  | 4 | 4.00 | 0.22 | 94.12 |
| 3.1 Describe different national models or health systems for provision of health care and their respective effects on health and health care expenditure. | 4 | 4.31 | 0.18 | 97.30 |  | 4 | 3.70 | 0.26 | 89.19 |
| 3.2 Describe how global trends in health care practice, commerce and culture, multinational agreements, and multinational organizations contribute to the quality and availability of health and health care locally and internationally. | 4 | 4.09 | 0.18 | 100.00 |  | 3 | 3.54 | 0.25 | 89.19 |
| 3.3 Describe the impact of R&D, production and access on global health from a global public health product perspective, and be aware of the intellectual property rights of health technologies | 4 | 3.80 | 0.20 | 97.30 |  | 4 | 3.57 | 0.28 | 89.19 |
| DOMAIN: 4. Determinants of Health (Social, Environmental, and Behavioral) Understand that social, economic, and environmental factors are important determinants of health, and that health is more than the absence of disease. | 5 | 4.72 | 0.13 | 100.00 |  | 5 | 4.38 | 0.17 | 100.00 |
| 4.1 Describe how cultural context and education influences perceptions of health and disease. | 5 | 4.40 | 0.20 | 97.14 |  | 4 | 4.17 | 0.22 | 94.29 |
| 4.2 List major social and economic determinants of health and their effects on the access to and quality of health services and on differences in morbidity and mortality between and within countries. | 5 | 4.65 | 0.13 | 100.00 |  | 5 | 4.38 | 0.19 | 100.00 |
| 4.3 Describe the relationship between access to and quality of water, sanitation, food, and air on individual and population health. | 5 | 4.39 | 0.19 | 94.59 |  | 4 | 4.03 | 0.24 | 91.89 |
| 4.4 Describe the behavioral factors of health determinants. E.g., incidence of HIV/AIDS vs. drug use, male homosexual behavior, and multiple sexual partners, chronic disease vs. smoking and lack of exercise, etc. | 5 | 4.34 | 0.20 | 97.30 |  | 4 | 4.19 | 0.20 | 94.59 |
| DOMAIN: 5. Capacity Strengthening. Capacity strengthening is sharing knowledge, skills, and resources for enhancing global public health programmes, infrastructure, and workforce to address current and future global public health needs. | 5 | 4.60 | 0.15 | 100.00 |  | 4 | 4.13 | 0.22 | 94.44 |
| 5.1 Collaborate with a host or partner organization to assess the organization’s operational capacity upon request. | 5 | 4.59 | 0.13 | 100.00 |  | 4 | 4.03 | 0.20 | 97.30 |
| 5.2 Cocreate strategies with the community to strengthen community capabilities, and contribute to reduction in health disparities and improvement of community health. | 5 | 4.51 | 0.14 | 100.00 |  | 4 | 3.59 | 0.25 | 89.19 |
| 5.3 In the case of being empowered, integrate community assets and resources to improve the health of individuals and populations. | 5 | 4.41 | 0.19 | 97.30 |  | 4 | 3.54 | 0.29 | 89.19 |
| DOMAIN: 6. Collaboration, Partnering, and Communication. Collaborating and partnering is the ability to select, recruit, and work with a diverse range of global health stakeholders to advance research, policy, and practice goals, and to foster open dialogue and effective communication with partners and within a team. | 5 | 4.89 | 0.07 | 100.00 |  | 4 | 4.26 | 0.17 | 97.22 |
| 6.1 Include representatives of diverse constituencies in community partnerships and foster interactive learning with these partners. Communicate joint lessons learned to community partners and global constituencies. | 5 | 4.58 | 0.14 | 100.00 |  | 4 | 4.19 | 0.17 | 100.00 |
| 6.2 Demonstrate diplomacy and build trust with community partners. Exhibit inter professional values and communication skills that demonstrate respect for, and awareness of, the unique cultures, values, roles/responsibilities and expertise represented by other professionals and groups that work in global health. | 5 | 4.65 | 0.12 | 100.00 |  | 4 | 3.92 | 0.21 | 94.59 |
| 6.3 Apply leadership practices that support collaborative practice and team effectiveness. | 4 | 4.36 | 0.16 | 97.30 |  | 4 | 3.70 | 0.23 | 91.89 |
| 6.4 Ability to spread knowledge and skills. | 5 | 4.50 | 0.15 | 97.30 |  | 4 | 4.14 | 0.22 | 91.67 |
| DOMAIN: 7. Ethics, Health Equity and Social Justice. Encompasses the application of basic principles of ethics to global health issues and settings. Health equity and social justice is the framework for analyzing strategies to address health disparities across socially, demographically, or geographically defined populations. | 5 | 4.75 | 0.11 | 100.00 |  | 4 | 3.96 | 0.24 | 94.29 |
| 7.1 Demonstrate an understanding of and an ability to resolve common ethical issues and challenges that arise when working within diverse economic, political, and cultural and religious contexts as well as when working with vulnerable populations and in low-resource settings to address global health issues. Apply social justice and human rights principles in addressing global health problems | 5 | 4.64 | 0.13 | 100.00 |  | 4 | 3.81 | 0.24 | 91.89 |
| 7.2 Demonstrate an awareness of local and national codes of ethics relevant to one’s working environment. | 5 | 4.72 | 0.11 | 100.00 |  | 4 | 4.15 | 0.21 | 94.59 |
| 7.3 Apply the fundamental principles of international standards for the protection of human subjects in diverse cultural settings. | 5 | 4.65 | 0.13 | 100.00 |  | 4 | 3.84 | 0.24 | 89.19 |
| 7.4 Understand the barriers to access and equity of primary health care services for populations in developing countries. | 5 | 4.51 | 0.14 | 100.00 |  | 4 | 4.09 | 0.22 | 91.89 |
| 7.5 Implement strategies to engage marginalized and vulnerable populations in making decisions that affect their health and well-being. | 5 | 4.41 | 0.16 | 100.00 |  | 4 | 3.62 | 0.24 | 91.89 |
| 7.6 Demonstrate a basic understanding of the relationships between health, human rights, and global inequities. | 4 | 4.19 | 0.21 | 97.30 |  | 4 | 3.97 | 0.24 | 91.89 |
| 7.7 Demonstrate a commitment to social responsibility. | 5 | 4.64 | 0.13 | 100.00 |  | 4 | 4.00 | 0.24 | 91.43 |
| DOMAIN: 8. Professional Practice. Refers to activities related to the specific profession or discipline of the global health professional. | 5 | 4.82 | 0.08 | 100.00 |  | 5 | 4.53 | 0.12 | 100.00 |
| 8.1 Demonstrate the ability to adapt discipline-specific skills and practice in are source-constrained setting. | 5 | 4.58 | 0.12 | 100.00 |  | 4 | 4.00 | 0.22 | 94.59 |
| 8.2 Capabilities of participation in national health security, including global health and safety assessment, practice, emergency response and other capabilities. | 5 | 4.35 | 0.19 | 97.30 |  | 4 | 3.95 | 0.22 | 97.30 |
| DOMAIN: 9. Programme Management. Programme management is ability to design, implement, and evaluate global health programmes to maximize contributions to effective policy, enhanced practice, and improved and sustainable health outcomes. | 5 | 4.75 | 0.11 | 100.00 |  | 4 | 4.36 | 0.15 | 100.00 |
| 9.1 Strategic analysis. According to the health determinants, conduct a need assessment and situation analysis across a range of cultural, economic, and health contexts. | 5 | 4.58 | 0.13 | 100.00 |  | 4 | 4.23 | 0.19 | 97.30 |
| 9.2 Design context-specific health interventions based on situation analysis, implement, and evaluate an evidence-based programme. | 5 | 4.69 | 0.11 | 100.00 |  | 4 | 4.07 | 0.18 | 97.30 |
| 9.3 Apply project management techniques throughout programme planning, implementation, and evaluation. | 5 | 4.65 | 0.13 | 100.00 |  | 4 | 4.23 | 0.22 | 94.59 |
| 9.4 Identify methods for assuring programme sustainability. | 5 | 4.59 | 0.13 | 100.00 |  | 4 | 3.69 | 0.23 | 94.44 |
| DOMAIN: 10. Sociocultural, Political Awareness and Policy Promotion. Sociocultural and political awareness is the conceptual basis with which to work effectively within diverse cultural settings and across local, regional, national, and international political landscapes. | 5 | 4.49 | 0.13 | 100.00 |  | 4 | 3.92 | 0.20 | 100.00 |
| 10.1 Describe the roles and relationships of the major entities influencing global health and development; describe the various global health actors, the role of different types of actors in global health governance, their contribution and challenges, and coping strategies. | 4 | 4.09 | 0.17 | 100.00 |  | 4 | 3.74 | 0.21 | 97.30 |
| 10.2 Describe China's basic national conditions, history of global health activities, experience and lessons. China's role and its functions, and policies in global health under new situations. | 5 | 4.66 | 0.11 | 100.00 |  | 4.5 | 4.38 | 0.16 | 100.00 |
| 10.3 Awareness of the information of politics, culture, environment, society, religion, law, diplomacy and national security. | 5 | 4.62 | 0.14 | 97.30 |  | 4 | 3.92 | 0.23 | 94.59 |
| 10.4 The ability to shape policy. Familiar with the policy procedures and political characteristics of the target country, with the ability to translate data, evidence and work plans into policy statements, policy documents and the implementation of relevant policies in a complex policy environment. | 5 | 4.38 | 0.18 | 97.30 |  | 4 | 3.65 | 0.23 | 94.59 |
| DOMAIN: 11. Personal Competencies | 5 | 4.76 | 0.09 | 100.00 |  | 4 | 4.40 | 0.15 | 100.00 |
| 11.1 Communicate effectively in the official language of the target context and the ability to work cross-culturally. | 5 | 4.81 | 0.10 | 100.00 |  | 4 | 4.19 | 0.22 | 94.59 |
| 11.2 Acknowledge one’s limitations in skills, knowledge, and abilities. Fast learning. Get the most up-to-date and comprehensive information quickly; quickly locate the knowledge points you need for your work from the vast amount of information | 5 | 4.65 | 0.13 | 100.00 |  | 4 | 4.12 | 0.18 | 97.30 |
| 11.3 Emotion management (compression and conflict response) skills. No personality defects, strong psychological endurance and coping skills | 5 | 4.78 | 0.10 | 100.00 |  | 4 | 4.01 | 0.19 | 94.59 |
| 11.4 Demonstrate integrity, regard, and respect for others in all aspects of professional practice. | 5 | 4.88 | 0.08 | 100.00 |  | 5 | 4.43 | 0.20 | 97.30 |

Results of the panel discussion

Issue 1: Should the word ‘Describe’ be replaced by ‘analyze’, ‘use’, ‘known’, or ‘understand’? (e.g. Competency 1, 3.3, 4.1, 4.3)

Consensus: ‘Describe’ is a way to measure the level of ‘understanding’; ‘Understand/know’ the general concepts are basic requirement for all, while to ‘use’ or ‘analyze’ (or even critically analyze) are advanced requirements for whose specialized field. This framework should focus on the basic competencies that are needed for all public health professionals.

Issue 2: Is there a need to limit the scope of the competencies?

For ‘1.1 Ability to analyze the morbidity and mortality of major disease around the world, and the indicators and trends of disease burden’, an expert commented in the 3^rd^ round consultation that the scope of ‘major diseases’ was overly broad and it could be limited to certain diseases, i.e. tropical diseases.

Consensus: It is important for all public health professionals to follow up with the major diseases with an understanding of the core knowledge, while some may have a higher level of the ability to analyze in his/her own field.

For ‘2.1-2.3 Describe the major global health initiatives, the major global health efforts, include main activities, financing and stakeholders, and major intervention strategies, best practices, limitation of current interventions, and innovation strategies of public health issues’, an expert suggested in the 3^rd^ round consultation that to add an adjunct ‘relating with his/her own professional field’.

Consensus: Replace ‘Describe’ with ‘Understand’; all the public health professionals should have an understanding of the important health initiatives and efforts, which is a basic competency; while regarding his/her own professional field, he/she should be able to analyze, apply, implement these initiatives, efforts, or intervention strategies—which is a higher level of competency.

For ‘2.4 Describe the global health history and its development, especially the work history of developing countries and its current situation, and the ability to analyze and learn from the past’, an expert suggested in the 3^rd^ round consultation that ‘The global health history and its development’ was overly broad.

Consensus: Keep as it is.

For ‘3.1 Describe different national models or health systems for provision of health care and their respective effects on health and health care expenditure’, an expert suggested in the 3^rd^ round consultation that ‘different national…’ could be revised to ‘Major countries with geographical and disease representation’ or ‘target countries’.

Consensus: Revise ‘different national’ to ‘Typical countries’ or ‘Representative countries’. For all public health professionals, it is a basic competency to understand or acquainted with the typical health system styles, while to ‘analyze the effects on health and health care expenditure’ are the advanced requirements for those who work in health systems and service deliveries.

Issue 3: How to better summarize the following competencies?

For ‘3.3 Describe the impact of R&D, production and access on global health from a global public health product perspective, and be aware of the intellectual property rights of health technologies’, an expert suggested in the 3^rd^ round consultation that to better present this competency from the perspective of health technology, products, and economics (their relationship and influence) or intellectual property could even be listed as a solely competency.

Consensus: ‘Describe the impact of R&D, production and access on global health from a global public health product perspective’ is relatively specialized which is only feasible for those who are work in health economy or health technology assessment; while the latter sentence ‘be aware of the intellectual property rights of health technologies’ should be the basic competency for all. Thus, the latter sentence was kept.

For ‘6.2 Demonstrate diplomacy and build trust with community partners. Exhibit inter-professional values and communication skills that demonstrate respect for, and awareness of, the unique cultures, values, roles/responsibilities and expertise represented by other professionals and groups that work in global health’, an expert suggested in the 3^rd^ round consultation that communication and respect could be broken down into two competencies.

Consensus: Communication skills are important, and it could be combined with 6.4 ‘Ability to spread knowledge and skills’. Proposed text: Exhibit inter-professional values and communication skills that demonstrate respect for, and awareness of, the unique cultures, values, roles/responsibilities and expertise represented by other professionals and groups that work in global health.

Issue 4: Regarding the competencies that not specifically serves for global health, but also applies to other competencies, should they be kept?

6.4 Ability to spread knowledge and skills.

Consensus: refer to 6.2.

11.2 Acknowledge one’s limitations in skills, knowledge, and abilities. Fast learning. Get the most up-to-date and comprehensive information quickly; quickly locate the knowledge points you need for your work from the vast amount of information.

Consensus: Remove this requirement.

11.4 Demonstrate integrity, regard, and respect for others in all aspects of professional practice.

Consensus: Keep this requirement. Integrity is an important component of global health, which supports cooperation.

Issue 5: How to improve the organization of among secondary competencies of the secondary competencies under ‘DOMAIN: 6. Collaboration, Partnering, and Communication’ as an expert commented in the 3^rd^ round consultation that the secondary competencies were poorly organized in logic.

Consensus: Organized by the following logic: 1. Respect & culturally sensitive; 2. Communication & dissemination; 3. Leadership & efficiency.

Issue 6: How to improve the feasibility of the competencies?

5.2 Co-create strategies with the community to strengthen community capabilities, and contribute to reduction in health disparities and improvement of community health.

Consensus: There is no necessary causal link between improving personnel capacity strategies and reducing unfairness. It is recommended to delete “and thus...”.

6.3 Apply leadership practices that support collaborative practice and team effectiveness.

Consensus: This competency is difficult to measure by quantitative methods, but it is important to have. Qualitative methods can be used.

All of the competencies under ‘DOMAIN: 9. Programme Management’.

Consensus: To simplify and keep the basic skills of project management, change 9.1 to ability of design (needs and evidence), 9.2 to ability implementation, 9.3 to ability of supervise and evaluation (sustainability). ‘Local ownership’ should be considered in all competencies under DOMAIN 9. 9.1 project design should involve local personnel; 9.2 implementation should be the ability to organize and mobilize local personnel to implement projects. 9.3 supervisory assessments should include the influence on local policy improvements to promote sustainability of outcomes.

Other changes according to the panel discussion include: 1) Suggested to rearrange domains in logical order: knowledge-skills-practice. 2) To unify and standardize the language.
