# Supplementary material for: Adaptation of the CUGH global health competency framework in the Chinese context: a mixed-methods study
Source: Glob Health Res Policy. 2023 Nov 2;8:46. doi: 10.1186/s41256-023-00327-w (PMC10621075; doi:10.1186/s41256-023-00327-w)
Supplement: Supplementary file 9 — Additional file 9: Full version of the adapted CUGH global health competencies in Chinese context. [file 41256_2023_327_MOESM9_ESM.docx]

**Additional file 9. Full version of the adapted CUGH global health competencies in Chinese context**

| **1. 全球疾病负担**  **Global Burden of Disease**  了解高、中、低收入国家和地区的主要疾病负担的分布及原因。  Understand the distribution and causes of major disease burden in high-, middle- and low-income countries, territories and areas. |
| --- |
|  |
| 1.1了解全球主要疾病的发病和死亡原因^[[1]](#footnote-1)^、疾病负担指标^[[2]](#footnote-2)^及其变化趋势。  Understand the morbidity and mortality of major disease around the world^1^, and the indicators^2^ and trends of disease burden. |
| 1.2能够通过获取和应用公开资料和数据^[[3]](#footnote-3)^了解特定人群的疾病和健康信息。  Ability to acquire disease and health information of target population through public literature and data^3^. |
| 1.3能够分析全球卫生领域重要热点问题或挑战^[[4]](#footnote-4)^。  Ability to analyze key issues or challenges in the global health arena^4^. |
| **2．影响健康的社会经济、环境和行为因素**  **Socioeconomic, Environmental and Behavioral Determinants of Health**  了解社会、经济、环境和行为是健康的重要影响因素及其之间的相互作用，健康不仅是没有疾病，健康在所有相关政策中有所体现。  Understand that social, economic, environmental and behavioral factors, along with their interactions, are important determinants of health. Health is more than the absence of disease, which should be considered in all policies. |
| 2.1了解文化和宗教背景、受教育程度如何影响人们对健康和疾病的认识。  Understand how cultural context, religion and education influence perceptions of health and disease. |
| 2.2能够列出影响健康的主要社会^[[5]](#footnote-5)^和经济因素，及其对医疗卫生服务可及性和质量的影响。  List major social^5^ and economic determinants of health and their effects on the access to and quality of health services. |
| 2.3知晓饮用水、食品、卫生条件、空气、土壤以及医疗设施的可及性与质量对个体和人群健康的影响。  Understand the relationship between access to and quality of water, food, sanitation, air, earth and health facilities on individual and population health. |
| 2.4能够描述影响健康的主要个体行为因素^[[6]](#footnote-6)^。  The ability to describe the behavioral factors of health determinants^6^. |
| **3. 全球化对人群健康、卫生系统和医疗服务的影响**  **The Impact of Globalization on Population Health, Health Systems and Healthcare**  了解全球化如何影响人群健康、卫生系统和医疗服务。  Understand how globalization affects health, health systems and health care |
| 3.1能够描述典型国家卫生体系类型或医疗服务模式，及其对健康和卫生支出的影响。  Describe typical national healthcare systems or healthcare service models and their impacts on health and health care expenditure. |
| 3.2能够描述全球化进程中商贸、文化、医疗卫生等因素对本地和全球健康和医疗卫生服务/产品的影响^[[7]](#footnote-7)^。  Describe the impact of commerce, culture, health and other factors on local and global health care, while taking into account globalization^7^. |
| 3.3了解知识产权制度对包括药品在内的卫生技术研发创新的激励作用和局限性。  Understand the incentives and limitations of Intellectual Property system for health technology Research & Development (R&D), including pharmaceuticals. |
| **4．全球卫生领域的重要倡议和行动**  **Major Global Health Initiatives and Efforts**  了解全球卫生的历史和重要倡议，能够辩证地思考全球卫生优先领域的演变以及当前的全球卫生行动。  Knowledge of global health history and major initiatives, and the ability to think critically about the changing priorities on global health issues and current global health efforts. |
| 4.1了解重要的全球卫生倡议^[[8]](#footnote-8)^。  Knowledge of major global health initiativies^8^. |
| 4.2了解全球重要卫生行动^[[9]](#footnote-9)^。  Knowledge of major global health efforts^9^. |
| 4.3了解全球主要疾病和卫生问题的主要干预策略。  Knowledge of major diseases around the world and major intervention strategies of public health issues. |
| 4.4了解全球卫生的历史和现状，能够分析经验和教训。  Knowledge of global health history and its current situation, and the ability to analyze and learn from the past. |
| **5.伦理、卫生公平和社会正义**  **Ethics, Health Equity and Social Justice**  具备在运用基本伦理准则处理全球卫生问题的能力；具备运用卫生公平和社会正义分析框架处理不同社会环境、人口学或地理学特征人群所面对的健康不公平问题的能力。  Ability to address global health issues with the basic principles of ethics; ability to address health disparities by health equity and social justice frameworks across socially, demographically, or geographically defined populations. |
| 5.1在不同经济、政治、文化和宗教背景下工作时，或与弱势群体工作时，能够判断卫生项目是否能够符合当地的伦理规范，理解并能够制定出符合基本伦理准则和适合当地情境的解决方案。  Ability to identify whether health projects are in accordance with local ethics, to resolve common ethical issues and challenges that arise when working within diverse economic, political, cultural and religious contexts as well as when working with vulnerable populations. |
| 5.2具备与工作环境相关的地方和国家道德规范的意识。  Awareness of local and national codes of ethics relevant to one’s working environment. |
| 5.3能够运用国际标准中的基本原则^[[10]](#footnote-10)^来保护不同宗教、文化背景下的脆弱人群。  Apply the fundamental principles of international standards^10^ for the protection of human subjects in diverse cultural settings. |
| 5.4理解和认识发展中国家人口获得初级卫生保健服务的可及性和公平性的障碍。  Understand the barriers to access and equity of primary health care services for populations in developing countries. |
| 5.5能够应用卫生公平策略促使边缘和弱势群体参与影响其健康和福祉的决策。  Implement strategies to engage marginalized and vulnerable populations in making decisions that affect their health and well-being. |
| 5.6基本理解健康差异、人权和全球不公平之间的关系。  Demonstrate a basic understanding of the relationships between health disparities, human rights, and global inequities. |
| 5.7具备良好的社会责任感^[[11]](#footnote-11)^。  Demonstrate a commitment to social responsibility^11^. |
| **6.社会文化、政治意识和政策推动**  **Sociocultural, Political Awareness and Policy Promotion**  社会文化和政治意识是在不同文化背景下，在地方、区域、国家和国际政治环境中有效工作的重要前提。  Sociocultural and political awareness is the conceptual basis with which to work effectively within diverse cultural settings and across local, regional, national and international political landscapes. |
| 6.1 能够描述影响全球卫生发展的主体之间的角色及其关系，描述全球卫生行为体的多元化、不同类别行为体在全球卫生治理中的作用、贡献和面临的挑战以及应对策略。  Describe the roles and relationships among actors that influence global health development, describe the various global health actors, the role of different types of actors in global health governance, their contribution and challenges, and coping strategies. |
| 6.2能够描述中国基本国情、中国的全球卫生角色、地位和作用，以及新形势下中国开展全球卫生工作的主要方针和政策。  Describe China's basic national conditions, roles, and policies in global health under new situations. |
| 6.3对体制、文化、环境、社会、宗教、法律^[[12]](#footnote-12)^、外交、国家安全等领域的信息具有敏感性。  Awareness of the information of politics, culture, environment, society, religion, law^12^, diplomacy and national security. |
| 6.4熟知工作国的政策程序和政治特征，具有在复杂的政策环境下将数据、证据和方案转换成政策表述、政策文件以及将相关政策推动落实的能力。  Familiar with the policy procedures and political characteristics of the target country, with the ability to translate data, evidence and work plans into policy statements, policy documents and the implementation of relevant policies in a complex policy environment. |
| **7.** **与全球卫生相关的个人基本素养和专业实践积累**  **Personal Competencies and Professional Practice**  具备自身专业或学科有关活动所需要的必备素养、知识、技能和实践经验。  The necessary competencies, knowledge, skills and practical experience needed for professional activities. |
| 7.1 能够运用工作环境的官方语言进行有效沟通，跨文化开展工作。  Communicate effectively in the official language of the target context and the ability to work cross-culturally. |
| 7.2具备情绪管理能力、强大的心理承受能力和应对、解决冲突的技巧与能力。  Emotion management skills, strong psychological endurance and skills and abilities to cope with and resolve conflicts. |
| 7.3在专业实践的各个方面显示出诚信。  Demonstrate integrity in all aspects of professional practice. |
| 7.4具有在资源有限的环境中开展专业技术工作的能力。  Ability to apply discipline-specific skills and practice in a resource-constrained setting. |
| **8.能力加强**  **Capacity Strengthening**  能力加强是指通过分享知识、技能和资源、完善全球公共卫生项目和基础设施、促进人力资源培养，来解决目前和未来的全球公共卫生需求^[[13]](#footnote-13)^。  Capacity strengthening is sharing knowledge, skills and resources for enhancing global public health programmes, infrastructure and workforce to address current and future global public health needs.^13^ |
| 8.1能够与合作方共同评估卫生服务提供机构的卫生服务提供能力，识别差距，提出有针对性的建议。  Collaborate with a host or partner organization to assess the organization’s operational  Capacity, identify gaps and propose corresponding recommendations. |
| 8.2能够在跨国界/跨文化的情况下，与社区合作制定提升人员能力的策略和具体措施。  Cocreate strategies with the community to strengthen community capabilities, in a cross-border or cross cultural context. |
| 8.3能够从优化配置的角度，向合作机构和社区提供资源整合的依据和建议。  Integrate community assets and resources to improve the health of individuals and populations. |
| **9.合作与沟通**  **Collaboration, Partnering and Communication**  合作伙伴关系是为了改善人群健康，与各类全球卫生利益相关者开展合作，从而推动研究、影响卫生实践和政策制定的能力，以及与合作伙伴和团队内部建立开放式对话和有效沟通的能力。  Collaborating and partnering is the ability to select, recruit and work with a diverse range of global health stakeholders to advance research, policy and practice goals, and to foster open dialogue and effective communication with partners and within a team. |
| 9.1 具备跨学科视角和文化敏感性，尊重和理解从事全球卫生工作的专业人士和团体所代表的独特文化、价值观、角色/职责和专业。  Exhibit interprofessional values and communication skills that demonstrate respect for, and awareness of, the unique cultures, values, roles/responsibilities and expertise represented by other professionals and groups that work in global health. |
| 9.2具有与不同文化背景的合作伙伴良好的沟通技巧和传播的能力。  Demonstrate communication skills and information dissemination skills with partners from different cultural backgrounds. |
| 9.3能够运用领导力来开展合作和提升团队效率^[[14]](#footnote-14)^。  Apply leadership practices that support collaborative practice and team effectiveness^14^. |
| **10.全球卫生项目管理**  **Programme Management**  项目管理能力包括设计、实施、督导和评估全球卫生项目，以最大程度促进全球卫生政策的可及性、有效性、可持续地改善卫生服务、促进健康。  Programme management is ability to design, implement, supervise and evaluate global health programmes to maximize contributions to effective policy, enhanced practice, and improved and sustainable health outcomes. |
| 10.1具备项目设计能力，能够与当地人员共同基于循证原则对当地人群健康需求进行评估和策略分析^[[15]](#footnote-15)^。  Plan project, collaborate with local personnel, to analyze the health needs of target populations by evidence-based principles^15^. |
| 10.2具备组织项目实施能力^[[16]](#footnote-16)^，能够应用项目管理技能，因地制宜实施项目或开展干预措施。  Implement project^16^, apply project management skills, implement interventions according to local conditions. |
| 10.3具备项目督导评估能力，促进项目可持续发展^[[17]](#footnote-17)^。  Supervise and evaluate project to promote sustainable development of the project^17^. |

1. 包括时间、空间和人间三间分布，以及疾病风险如何因地因人群而异。

   Including the distribution of time, place and person, as well as how the risk of diseases various among location and population. [↑](#footnote-ref-1)
2. 各领域有代表性的疾病或卫生问题（特别是影响面广、受全球性关注的、列入SDG的）的疾病负担指标，例如传染病中的疟疾、艾滋病和结核等。

   Disease burden indicators for major health problems, especially those are wide-influencing, global-concerned, and listed in SDGs, such as malaria, HIV/AIDS, and tuberculosis. [↑](#footnote-ref-2)
3. 例如：国家、地区、国际组织等发布的公共卫生监测数据、健康统计数据、重要统计报告、专题调查或研究数据、卫生规划数据、政策文献、学术文献等。

   E.g. public health surveillance data published by nations, regions and international organizations, health statistics, health reports, thematic surveys or research data, electronic health records, health plan data, policy, and scientific literature. [↑](#footnote-ref-3)
4. 例如：新发传染病、生殖健康、妇幼卫生、慢性非传染性疾病和精神健康等领域问题，以及脆弱人群的主要健康问题。

   E.g. emerging infectious diseases, reproductive health, maternal and child health, non-communicable diseases and mental health, as well as major health issues of vulnerable populations. [↑](#footnote-ref-4)
5. 健康问题社会决定因素是指人们出生、生长、生活、工作和老年环境，包括卫生系统。这些环境受到全球、国家和地方各级金钱、权力和资源分配状况制约，并受政策选择的影响。健康问题社会决定因素是造成卫生不公平现象的主要因素，导致本可避免的国家内部以及国与国之间不公平的健康差异。（<https://www.who.int/topics/social_determinants/zh/>）

   The social determinants of health are the conditions in which people are born, grow, live, work and age. These circumstances are shaped by the distribution of money, power and resources at global, national and local levels. The social determinants of health are mostly responsible for health inequities - the unfair and avoidable differences in health status seen within and between countries. ( https://www.who.int/social_determinants/sdh_definition/en/) [↑](#footnote-ref-5)
6. 例如：合理的膳食习惯；慢病与吸烟、缺乏运动等相关；艾滋病发病与吸毒、男男性行为、多性伴行为等密切相关。

   E.g. dietary habit, chronic disease vs. smoking and lack of exercise, incidence of HIV/AIDS vs. drug use, male homosexual behavior, and multiple sexual partners. [↑](#footnote-ref-6)
7. 包括了解人口流动和贸易交流影响传染病的传播和慢性病的发展趋势；了解全球范围内医疗卫生工作者的可获得性和流动性的总体趋势以及发展中国家卫生人力资源危机。

   Including understand how population movement and trade contribute to the spread of communicable and the trend of chronic diseases; and understand the trends in the global availability and movement of

   health care workers and the health care workforce crisis in the developing world. [↑](#footnote-ref-7)
8. 例如：联合国2030年可持续发展议程中与健康相关的主要目标和指标、每年世界卫生大会的重要决议等。

   E.g., Health targets in the 2030 Agenda for Sustainable Development, and important resolutions of World Health Assembly. [↑](#footnote-ref-8)
9. 例如：抗艾滋病、结核和疟疾全球基金、全球疫苗免疫联盟等一系列全球卫生行为体所开展的卫生行动。具体内容包括了解全球重要卫生行动的目标、实施内容与策略、利益相关方与合作机制、面临的挑战及其筹融资情况等。

   Health efforts taken by global health actors, e.g. Global Fund to Fight AIDS, TB, and Malaria, the Global Alliance for Vaccines and Immunizations, include knowledge of the goals, main activities, strategies, stakeholder and cooperation mechanisms, challenges and financing of major global health efforts. [↑](#footnote-ref-9)
10. 伦理标准包括：《世界医学协会赫尔辛基宣言》、《人体生物医学研究国际伦理指南》、WHO相关伦理标准，及研究人所在机构的伦理委员会标准等；人权原则包括：联合国1948年通过的《世界人权宣言》及1966年通过的《经济、社会、文化权利国际公约》、《公民权利和政治权利国际公约》等。

    Ethical standards include: World Medical Association Declaration of Helsinki, International Ethical Guidelines for Biomedical Research Involving Human Subjects, the relevant ethical standards of the WHO, and the standards of the ethics committee of the institution where the researcher is affiliated; the human rights principles include: The Universal Declaration of Human Rights, which was adopted by the UN General Assembly on 10 December 1948 and the International Covenant on Economic, Social and Cultural Rights (ICESCR) and the International Covenant on Civil and Political Rights, adopted by the United Nations General Assembly on 16 December 1966. [↑](#footnote-ref-10)
11. 根据CUGH教学手册，本指标要求个人以自身专业作为社会责任参与地区、国家或国际的相关活动以提升政治意识，改善影响健康的社会因素。

    According to the CUGH competency tool kit, this indicator requires individuals to participate in regional, national or international activities with their own professionalism as social responsibility to enhance political awareness and improve social determinants that affect health. [↑](#footnote-ref-11)
12. 包括国际法和国际惯例、卫生法律法规和监督、WHO的组织法和IHR（2005）等。

    Including international law and international practices, health laws and regulations and supervision, WHO's organizational law and IHR (2005), etc. [↑](#footnote-ref-12)
13. 能力建设主体包括但不限于不同级别的卫生体系的能力、机构的能力、个体层面的能力。

    The main body of capacity building includes, but is not limited to, the capabilities of different levels of health systems, institutional capabilities, and individual level capabilities. [↑](#footnote-ref-13)
14. 包括组织工作团队积累专业、知识的能力。

    Including the ability of the organization team to accumulate professional and knowledge. [↑](#footnote-ref-14)
15. 策略分析是利用系统思维来分析影响健康趋势形成的各种复杂关联因素，从而在地方、国家和国际层面设计项目。

    Strategic analysis is the ability to use systems thinking to analyze a diverse range of complex and interrelated factors shaping health trends to formulate programs at the local, national, and international levels. [↑](#footnote-ref-15)
16. 即具备组织动员当地人员实施项目的能力。

    That is, the ability to organize and mobilize local personnel to implement projects. [↑](#footnote-ref-16)
17. 包括用督导评估的结果影响当地的政策改善，以促进层面成果的可持续性。

    This includes the use of supervisory assessments to influence local policy improvements to promote sustainability of outcomes. [↑](#footnote-ref-17)
