# Supplementary material for: Adaptation of the CUGH global health competency framework in the Chinese context: a mixed-methods study
Source: Glob Health Res Policy. 2023 Nov 2;8:46. doi: 10.1186/s41256-023-00327-w (PMC10621075; doi:10.1186/s41256-023-00327-w)
Supplement: Supplementary file 10 — Additional file 10: Comparison of the CUGH framework and the adapted CUGH framework in Chinese setting. [file 41256_2023_327_MOESM10_ESM.docx]

**Additional file 10. Comparison of the CUGH framework and the adapted CUGH framework in Chinese setting**

| THE CUGH FRAMEWORK^^[[1]](#endnote-1)^^  (program-oriented operational level under level III) | THE ADAPTED CUGH FRAMEWORK IN CHINESE SETTING |
| --- | --- |
| DOMAIN: 1. Global Burden of Disease.  Encompasses basic understandings of major causes of morbidity and mortality and their variations between high-, middle- and low-income regions, and with major public health efforts to reduce health disparities globally. | 1. Global Burden of Disease  Understand the distribution and causes of major disease burden in high-, middle- and low-income countries, territories and areas. |
| 1a. Describe the major causes of morbidity and mortality around the world, and how the risk for disease varies with regions. | 1.1 Understand the morbidity and mortality of major disease around the world^[[2]](#footnote-1)^, and the indicators and trends of disease burden^[[3]](#footnote-2)^. |
| 1b. Describe major public health efforts to reduce disparities in global health (such as Millennium Development Goals and Global Fund to Fight AIDS, TB, and Malaria). | 1.2 Ability to acquire disease and health information of target population through public literature and data^[[4]](#footnote-3)^. |
| 1c. Validate the health status of populations using available data (e.g., public health surveillance data, vital statistics, registries, surveys, electronic health records, and health plan claims data). | 1.3 Ability to analyze key issues or challenges in the global health arena^[[5]](#footnote-4)^. |
| DOMAIN: 2. Globalization of Health and Health Care.  Focuses on understanding how globalization affects health, health systems, and the delivery of health care. | 2．Social-economic, Environmental and Behavioral Determinants of Health  Understand that social, economic, environmental and behavioral factors, along with their interactions, are important determinants of health. Health is more than the absence of disease, which should be considered in all policies. |
| 2a. Describe different national models or health systems for provision of health care and their respective effects on health and health care expenditure. | 2.1 Understand how cultural context, religion and education influence perceptions of health and disease. |
| 2b. Describe how global trends in health care practice, commerce and culture, multinational agreements, and multinational organizations contribute to the quality and availability of health and health care locally and internationally. | 2.2 List major social^[[6]](#footnote-5)^ and economic determinants of health and their effects on the access to and quality of health services. |
| 2b. Describe how global trends in health care practice, commerce and culture, multinational agreements, and multinational organizations contribute to the quality and availability of health and health care locally and internationally. | 2.3 Understand the relationship between access to and quality of water, food, sanitation, air, earth and health facilities on individual and population health. |
| 2c. Describe how travel and trade contribute to the spread of communicable and chronic diseases. | 2.4 The ability to describe the behavioral factors of health determinants^[[7]](#footnote-6)^. |
| 2d. Describe general trends and influences in the global availability and movement of health care workers. |  |
| DOMAIN: 3. Social and Environmental Determinants of Health.  Focuses on an understanding that social, economic, and environmental factors are important determinants of health, and that health is more than the absence of disease. | 3. The Impact of Globalization on Population Health, Health Systems and Healthcare  Understand how globalization affects health, health systems and health care |
| 3a. Describe how cultural context influences perceptions of health and disease. | 3.1 Describe typical national healthcare systems or healthcare service models and their impacts on health and health care expenditure. |
| 3b. List major social and economic determinants of health and their effects on the access to and quality of health services and on differences in morbidity and mortality between and within countries. | 3.2 Describe the impact of commerce, culture, health and other factors on local and global health care, while taking into account globalization^[[8]](#footnote-7)^. |
| 3c. Describe the relationship between access to and quality of water, sanitation, food, and air on individual and population health. | 3.3 Understand the incentives and limitations of Intellectual Property system for health technology Research & Development (R&D), including pharmaceuticals. |
| DOMAIN: 4. Capacity Strengthening.  “Capacity strengthening is sharing knowledge, skills, and resources for enhancing global public health programs, infrastructure, and workforce to address current and future global public health needs.” | 4．Major Global Health Initiatives and Efforts  Knowledge of global health history and major initiatives, and the ability to think critically about the changing priorities on global health issues and current global health efforts. |
| 4a. Collaborate with a host or partner organization to assess the organization’s operational capacity. | 4.1 Knowledge of major global health initiatives ^[[9]](#footnote-8)^. |
| 4b. Cocreate strategies with the community to strengthen community capabilities, and contribute to reduction in health disparities and improvement of community health. | 4.2 Knowledge of major global health efforts^[[10]](#footnote-9)^. |
| 4c. Integrate community assets and resources to improve the health of individuals and populations. | 4.3 Knowledge of major diseases around the world and major intervention strategies of public health issues. |
| 4d. Identify methods for assuring program sustainability. (Proposed by members of the CUGH Global Health Competency Subcommittee.) | 4.4 Knowledge of global health history and its current situation, and the ability to analyze and learn from the past. |
| DOMAIN: 5. Collaboration, Partnering, and Communication.  “Collaborating and partnering is the ability to select, recruit, and work with a diverse range of global health stakeholders to advance research, policy, and practice goals, and to foster open dialogue and effective communication” with partners and within a team. | 5. Ethics, Health Equity and Social Justice  Ability to address global health issues with the basic principles of ethics; ability to address health disparities by health equity and social justice frameworks across socially, demographically, or geographically defined populations. |
| 5a. Include representatives of diverse constituencies in community partnerships and foster interactive learning with these partners. | 5.1 Ability to identify whether health projects are in accordance with local ethics, to resolve common ethical issues and challenges that arise when working within diverse economic, political, cultural and religious contexts as well as when working with vulnerable populations. |
| 5b. Demonstrate diplomacy and build trust with community partners.1 | 5.2 Awareness of local and national codes of ethics relevant to one’s working environment. |
| 5c. Communicate joint lessons learned to community partners and global constituencies. | 5.3 Apply the fundamental principles of international standards^[[11]](#footnote-10)^ for the protection of human subjects in diverse cultural settings. |
| 5d. Exhibit interprofessional values and communication skills that demonstrate respect for, and awareness of, the unique cultures, values, roles/responsibilities and expertise represented by other professionals and groups that work in global health. | 5.4 Understand the barriers to access and equity of primary health care services for populations in developing countries. |
| 5e. Acknowledge one’s limitations in skills, knowledge, and abilities. | 5.5 Implement strategies to engage marginalized and vulnerable populations in making decisions that affect their health and well-being. |
| 5f. Apply leadership practices that support collaborative practice and team effectiveness. | 5.6 Demonstrate a basic understanding of the relationships between health disparities, human rights, and global inequities. |
|  | 5.7 Demonstrate a commitment to social responsibility^[[12]](#footnote-11)^. |
| DOMAIN: 6. Ethics.  Encompasses the application of basic principles of ethics to global health issues and settings. | 6. Sociocultural, Political Awareness and Policy Promotion  Sociocultural and political awareness is the conceptual basis with which to work effectively within diverse cultural settings and across local, regional, national and international political landscapes. |
| 6a. Demonstrate an understanding of and an ability to resolve common ethical issues and challenges that arise when working within diverse economic, political, and cultural contexts as well as when working with vulnerable populations and in low-resource settings to address global health issues. | 6.1 Describe the roles and relationships among actors that influence global health development, describe the various global health actors, the role of different types of actors in global health governance, their contribution and challenges, and coping strategies. |
| 6b. Demonstrate an awareness of local and national codes of ethics relevant to one’s working environment. | 6.2 Describe China's basic national conditions, roles, and policies in global health under new situations. |
| 6c. Apply the fundamental principles of international standards for the protection of human subjects in diverse cultural settings. | 6.3 Awareness of the information of politics, culture, environment, society, religion, law^[[13]](#footnote-12)^, diplomacy and national security. |
|  | 6.4 Familiar with the policy procedures and political characteristics of the target country, with the ability to translate data, evidence and work plans into policy statements, policy documents and the implementation of relevant policies in a complex policy environment. |
| DOMAIN: 7: Professional Practice.  Refers to activities related to the specific profession or discipline of the global health practitioner. (Domain definition proposed by members of the CUGH Global Health Competency Subcommittee.) | 7. Personal Competencies and Professional Practice  The necessary competencies, knowledge, skills and practical experience needed for professional activities. |
| 7a. Demonstrate integrity, regard, and respect for others in all aspects of professional practice. | 7.1 Communicate effectively in the official language of the target context and the ability to work cross-culturally. |
| 7b. Articulate barriers to health and health care in low-resource settings locally and internationally. | 7.2 Emotion management skills, strong psychological endurance and skills and abilities to cope with and resolve conflicts. |
| 7c. Demonstrate the ability to adapt clinical or discipline-specific skills and practice in a resource-constrained setting. | 7.3 Demonstrate integrity in all aspects of professional practice. |
|  | 7.4 Ability to apply discipline-specific skills and practice in a resource-constrained setting. |
| DOMAIN: 8. Health Equity and Social Justice.  “Health equity and social justice is the framework for analyzing strategies to address health disparities across socially, demographically, or geographically defined populations.” | 8. Capacity Strengthening  Capacity strengthening is sharing knowledge, skills and resources for enhancing global public health programmes, infrastructure and workforce to address current and future global public health needs.^[[14]](#footnote-13)^ |
| 8a. Apply social justice and human rights principles in addressing global health problems. | 8.1 Collaborate with a host or partner organization to assess the organization’s operational  Capacity, identify gaps and propose corresponding recommendations. |
| 8b. Implement strategies to engage marginalized and vulnerable populations in making decisions that affect their health and well-being. | 8.2 Cocreate strategies with the community to strengthen community capabilities, in a cross-border or cross-cultural context. |
| 8c. Demonstrate a basic understanding of the relationships between health, human rights, and global inequities. | 8.3 Integrate community assets and resources to improve the health of individuals and populations. |
| 8d. Describe role of WHO in linking health and human rights, the Universal Declaration of Human Rights, International Ethical Guidelines for Biomedical Research Involving Human Subjects. |  |
| 8e. Demonstrate a commitment to social responsibility. |  |
| 8f. Develop understanding and awareness of the health care workforce crisis in the developing world, the factors that contribute to this, and strategies to address this problem. |  |
| DOMAIN: 9. Program Management.  “Program management is ability to design, implement, and evaluate global health programs to maximize contributions to effective policy, enhanced practice, and improved and sustainable health outcomes.” | 9. Collaboration, Partnering and Communication  Collaborating and partnering is the ability to select, recruit and work with a diverse range of global health stakeholders to advance research, policy and practice goals, and to foster open dialogue and effective communication with partners and within a team. |
| 9a. Plan, implement, and evaluate an evidence-based program. | 9.1 Exhibit interprofessional values and communication skills that demonstrate respect for, and awareness of, the unique cultures, values, roles/responsibilities and expertise represented by other professionals and groups that work in global health. |
| 9b. Apply project management techniques throughout program planning, implementation, and evaluation. | 9.2 Demonstrate communication skills and information dissemination skills with partners from different cultural backgrounds |
|  | 9.3 Apply leadership practices that support collaborative practice and team effectiveness^[[15]](#footnote-14)^. |
| DOMAIN: 10. Sociocultural and Political Awareness.  “Sociocultural and political awareness is the conceptual basis with which to work effectively within diverse cultural settings and across local, regional, national, and international political landscapes.” | 10. Programme Management  Programme management is ability to design, implement, supervise and evaluate global health programmes to maximize contributions to effective policy, enhanced practice, and improved and sustainable health outcomes. |
| 10a. Describe the roles and relationships of the major entities influencing global health and development. | 10.1 Plan project, collaborate with local personnel, to analyze the health needs of target populations by evidence-based principles^[[16]](#footnote-15)^. |
|  | 10.2 Implement project^[[17]](#footnote-16)^, apply project management skills, implement interventions according to local conditions. |
|  | 10.3 Supervise and evaluate project to promote sustainable development of the project^[[18]](#footnote-17)^. |
| DOMAIN: 11. Strategic Analysis.  “Strategic analysis is the ability to use systems thinking to analyze a diverse range of complex and interrelated factors shaping health trends to formulate programs at the local, national, and international levels.” |  |
| 11a. Identify how demographic and other major factors can influence patterns of morbidity, mortality, and disability in a defined population. |  |
| 11b. Conduct a community health needs assessment. |  |
| 11c. Conduct a situation analysis across a range of cultural, economic, and health contexts. |  |
| 11d. Design context-specific health interventions based on situation analysis. |  |

1. Jogerst K, Callender B, Adams V, Evert J, Fields E, Hall T, et al. Identifying interprofessional global health competencies for 21st-century health professionals. Ann Glob Health. 2015;81(2):239-47. [↑](#endnote-ref-1)
2. Including the distribution of time, place and person, as well as how the risk of diseases various among location and population. [↑](#footnote-ref-1)
3. Disease burden indicators for major health problems, especially those are wide-influencing, global-concerned, and listed in SDGs, such as malaria, HIV/AIDS, and tuberculosis. [↑](#footnote-ref-2)
4. E.g., public health surveillance data published by nations, regions and international organizations, health statistics, health reports, thematic surveys or research data, electronic health records, health plan data, policy, and scientific literature. [↑](#footnote-ref-3)
5. E.g., emerging infectious diseases, reproductive health, maternal and child health, non-communicable diseases and mental health, as well as major health issues of vulnerable populations. [↑](#footnote-ref-4)
6. The social determinants of health are the conditions in which people are born, grow, live, work and age. These circumstances are shaped by the distribution of money, power and resources at global, national and local levels. The social determinants of health are mostly responsible for health inequities - the unfair and avoidable differences in health status seen within and between countries. ( <https://www.who.int/social_determinants/sdh_definition/en/>) [↑](#footnote-ref-5)
7. E.g., dietary habit, chronic disease vs. smoking and lack of exercise, incidence of HIV/AIDS vs. drug use, male homosexual behavior, and multiple sexual partners. [↑](#footnote-ref-6)
8. Including understand how population movement and trade contribute to the spread of communicable and the trend of chronic diseases; and understand the trends in the global availability and movement of health care workers and the health care workforce crisis in the developing world. [↑](#footnote-ref-7)
9. E.g., Health targets in the 2030 Agenda for Sustainable Development, and important resolutions of World Health Assembly. [↑](#footnote-ref-8)
10. Health efforts taken by global health actors, e.g., Global Fund to Fight AIDS, TB, and Malaria, the Global Alliance for Vaccines and Immunizations, include knowledge of the goals, main activities, strategies, stakeholder and cooperation mechanisms, challenges and financing of major global health efforts. [↑](#footnote-ref-9)
11. Ethical standards include: World Medical Association Declaration of Helsinki, International Ethical Guidelines for Biomedical Research Involving Human Subjects, the relevant ethical standards of the WHO, and the standards of the ethics committee of the institution where the researcher is affiliated; the human rights principles include: The Universal Declaration of Human Rights, which was adopted by the UN General Assembly on 10 December 1948 and the International Covenant on Economic, Social and Cultural Rights (ICESCR) and the International Covenant on Civil and Political Rights, adopted by the United Nations General Assembly on 16 December 1966. [↑](#footnote-ref-10)
12. According to the CUGH competency tool kit, this indicator requires individuals to participate in regional, national or international activities with their own professionalism as social responsibility to enhance political awareness and improve social determinants that affect health. [↑](#footnote-ref-11)
13. Including international law and international practices, health laws and regulations and supervision, WHO's organizational law and IHR (2005), etc. [↑](#footnote-ref-12)
14. The main body of capacity building includes, but is not limited to, the capabilities of different levels of health systems, institutional capabilities, and individual level capabilities. [↑](#footnote-ref-13)
15. Including the ability of the organization team to accumulate professional and knowledge [↑](#footnote-ref-14)
16. Strategic analysis is the ability to use systems thinking to analyze a diverse range of complex and interrelated factors shaping health trends to formulate programs at the local, national, and international levels. [↑](#footnote-ref-15)
17. That is, the ability to organize and mobilize local personnel to implement projects. [↑](#footnote-ref-16)
18. This includes the use of supervisory assessments to influence local policy improvements to promote sustainability of outcomes. [↑](#footnote-ref-17)
